# PathoSafe: Sub-1 KB Out-of-Distribution Rejection and Cluster-Aware Certification for Point-of-Care Pathogen Biosensing

**DOI:** 10.3390/bios16090518

**Published:** 2026-09-17

**Authors:** Gaoming He, Mengdi Hou, Jianbo Huang, Longquan Chen, Qihuang Gao, Bitie Lan

**Affiliations:** 1 Guangxi Key Laboratory of Machine Vision and Intelligent Control, Wuzhou University, Wuzhou 543000, China; hegaoming@gxuwz.edu.cn (G.H.); chenlongquan@gxuwz.edu.cn (L.C.); gaoqihuang@gxuwz.edu.cn (Q.G.); lanbitie@gxuwz.edu.cn (B.L.); 2 School of Computer Application, Guilin University of Technology, Guilin 541004, China

**Keywords:** out-of-distribution benchmark, distribution-free risk control, conformal abstention, sub-1 KB microcontroller deployment, pathogen microscopy, malaria microscopy

## Abstract

A point-of-care pathogen-microscopy readout must signal when a sample falls outside what its detector was trained on. PathoSafe is an 816-byte int8-projection low-rank Mahalanobis out-of-distribution (OOD) head reading a frozen detector’s penultimate feature. It reaches a cross-site area under the receiver operating characteristic curve (AUROC) of 0.980 and multi-source AUROC of 0.964 (parasitic-egg-dominated), within 0.01 of a 16,896-byte full-precision dense baseline, and adds a measured 30.76 µs and 816 bytes of read-only memory (ROM) constants on an STM32H743 development board, so abstention adds only a small measured cost on-chip. The advantage is the trunk feature rather than the integer form. Certification is the harder problem and carries our central result: slide clustering silently breaks the standard independent-sample certificate, since distribution-free risk control assumes exchangeable samples that patch-clustered medical data do not supply. On a degraded-input risk certificate, the naive cell-level version holds on only 20% of leakage-free re-splits, whereas the correct slide-level one is valid at 0.266 with its width set by the slide count rather than by any bound we evaluate. The deployed threshold inherits this less severely. A Hoeffding–Bentkus budget gives an illustrative count of 38 independent slides under the stated assumptions (35 when five seeds are used), which is about 1.3× what this benchmark provides. We release that leakage-free slide-disjoint benchmark: an in-distribution malaria task with cross-site (BBBC041) and multi-source (SIPaKMeD, parasitic egg, white blood cells) regimes.

## 1. Introduction

Malaria and other microscopy-diagnosed infections remain a heavy global burden, with an estimated 263 million malaria cases and 597,000 deaths in 2023—about 95% of which were in the WHO African region [1]. Field diagnosis still relies on Giemsa-stained blood-smear microscopy, which demands trained microscopists and stable laboratory infrastructure that are chronically scarce in the most affected regions. Compact detectors deployable on sub-USD-60 microcontroller (MCU) microscopes are a pragmatic alternative. A 400 MHz Cortex-M7 with a commodity camera runs a 21 K-parameter stain-morphology-decoupled malaria detector [2] offline, which is what makes an unattended community health post conceivable as a deployment target. Recent sub-25 K detectors, including UltraLightSqueezeNet [3], show that clinically useful accuracy fits the Flash and static random-access memory (SRAM) envelope of commodity MCUs.

The setting is a specific one, and everything that follows depends on it. A health worker at a community post images a Giemsa-stained smear on a portable MCU microscope. Software upstream of this work segments each field into single cells, and a detector frozen at manufacture labels them, with no connectivity, no on-site retraining and no specialist at hand. Referral to expert microscopy is the only escalation available, so the device is useful as screening support under expert oversight rather than as an autonomous diagnosis. Section 3.1 states these assumptions in full.

An edge pathogen detector needs two primitives to be trusted: out-of-distribution (OOD) rejection and risk-controlled abstention. Softmax confidence is poorly calibrated in modern networks [4], and under deployment shift, it separates in- from out-of-distribution inputs weakly (Section 4.3), so a clinically usable detector must reject inputs that fall outside its training distribution and abstain, referring ambiguous cases to a specialist. Yet no public benchmark evaluates these primitives for compact pathogen microscopy. As we show, the natural way to certify abstention on such benchmarks is silently invalidated by slide clustering.

### 1.1. The Gap

Three problems compound. First, the de facto NIH malaria benchmark uses a per-cell random split, but its cells derive from roughly 200 patient slides. A per-cell split leaks slide-level staining and illumination signatures, inflating accuracy estimates and the per-cell risk certificate computed on it. Second, no existing benchmark couples a leakage-free slide-disjoint split with multi-pathogen OOD regimes and a sub-1 KB microcontroller-deployable budget. That budget is what the detector leaves over. The backbone reused here already occupies 23.5 KB of int8 weights and 816 ms per cell on an STM32H743 [2], so a trustworthiness layer has to be small enough to leave that deployment intact. The post hoc scores that are small enough turn out to be weak under site shift, and the OOD methods are strong enough to help exceed the budget. Third, distribution-free abstention guarantees from conformal prediction and risk control assume exchangeable calibration samples, which is an assumption that cells clustered within slides violate. The first and the third are facets of one cause. Cells within a slide are not independent.

An earlier finding redirected this paper toward abstention. Under the frozen-detector constraint, with no on-site retraining, no task-driven restoration front-end shows a consistent advantage over plain pixel reconstruction on degraded accuracy. The five-seed paired differences among the four strategies are of mixed sign and well inside the between-seed spread (Section 4.2). Confidence gating does not reach a trustworthy operating point at usable coverage on any of the three seeds, either. Plain pixel reconstruction already matches what a restoration front-end recovers, so restoration offers no consistent further gain under this constraint, and the residual risk is shift rather than degradation. We benchmark and certify abstention instead.

### 1.2. Contributions

We organize our contributions around one methodological result and two supporting deployment results. All are driven by the same constraint, a fixed sub-1 KB on-device budget for the trustworthy add-on, no retraining of the deployed detector, and realistic site- and pathogen-shift at inference time. The detection backbone is our own prior 21 K-parameter detector [2], which is reused unchanged. The findings are outlined below:

- A cluster-aware certification finding for distribution-free abstention on patch-clustered medical microscopy. This is our central result. Standard distribution-free risk control, whether conformal prediction, risk-controlling prediction sets (RCPSs), or Learn-then-Test, assumes exchangeable calibration cells, but cells from one patient slide are not independent. We bring cluster-robust inference to distribution-free risk control at the point where a medical benchmark issues its guarantee and show what it costs: ignoring the cluster structure silently invalidates the certificate, and we quantify the slide budget that restores it. On a degraded-input risk certificate, a naive cell-level version looks tight at 0.054 yet holds on only 20% of leakage-free re-splits. The statistically correct slide-level version is valid at 0.252 on that same calibration split and 0.266 as the conservative three-seed budget carried through this paper (Section 4.6). Its width is set by the number of calibration slides rather than by the estimator, since none of the concentration bounds we evaluate tightens it at the available ≈30 slides. A Hoeffding–Bentkus budget calculation puts the requirement at 38 independent slides, which is about 1.3× the post-disjoint NIH count. This gives a concrete dataset-budget deliverable for the field.
- A trustworthy-abstention benchmark for on-microcontroller pathogen microscopy under realistic deployment shift. No public benchmark measures whether a sub-1 KB on-device pathogen-microscopy detector correctly defers ambiguous inputs to a specialist when the input drifts off-distribution. We contribute one with a slide-disjoint split so no slide is shared between calibration and test and an in-distribution malaria task. It adds two deployment-relevant OOD regimes. Cross-site keeps the label space but changes the site and the *Plasmodium* species, using BBBC041. Multi-source spans cervical cytology, parasitic egg, and white-blood-cell inputs, which are not all different pathogens. Evaluation is five-seed with a held-out in-distribution calibration partition.
- PathoSafe is a sub-1 KB int8-projection OOD/abstention head deployable on pathogen-microscopy microcontrollers. On the microcontroller-class target, post hoc scores that fit the on-device budget are too weak, with softmax and energy reaching 0.782 and a 0.810 cross-site area under the receiver operating characteristic curve (AUROC). OOD methods that work at scale exceed the budget. We close this gap with an 816-byte int8-projection low-rank Mahalanobis head, which is read from the frozen detector’s penultimate feature under deployable fixed-scale arithmetic. Section 4.3 reports the leaderboard. Fitting OOD scoring into 816 bytes of int8-projection arithmetic with floating-point score assembly and no retraining of the detector is what turns a server-side OOD score into a deployable one. On  an STM32H743 development board, this head adds a measured 30.76 µs, 816 bytes of read-only memory (ROM) constants, and 256 bytes of SRAM at 400 MHz; the kernel is faithful to the off-device reference within $2.3\times 10^{-5}$ (Section 4.7). The  deployment significance is interpreted in Section 5.

Two caveats qualify these results, which are detailed in Section 4.8. The cross-site number is a single deterministic point read from a training-seed-invariant frozen feature, and the reference trades a small amount of multi-source AUROC for that cross-site gain. The contribution is the specific combination of leakage-free splitting, multi-pathogen microscopy, a sub-1 KB integer budget, and finite-sample risk-controlled abstention, which to our knowledge no prior work brings together (Section 2).

The remainder is organized as follows. Section 2 reviews compact pathogen detection, OOD detection in medical imaging, distribution-free risk control, and data leakage in medical benchmarks. Section 3 specifies the protocol, the reference system, and the certification procedure. Section 4 presents the leaderboard, the cluster-aware certificate analysis, and the on-chip measurement. Section 5 discusses the caveats and limitations; Section 6 concludes.

## 2. Related Work

### 2.1. MCU-Deployable Pathogen Microscopy

Automated malaria microscopy is surveyed by Poostchi et al. [5] and Mujahid et al. [6]. Smartphone convolutional neural network (CNN) detection at the point of care is feasible [7,8]. The 2018–2024 literature converged on 20–100 K-parameter convolutional networks for MCU deployment, with UltraLightSqueezeNet [3] reaching sub-10 K by pruning, alongside smartphone-scale detectors [9]. The 21 K stain-morphology-decoupled backbone from which we read features [2] exemplifies this regime. Across the detectors surveyed above, evaluation is on clean laboratory images and splitting is per-cell and random, the backbone reused here being the exception. We are not aware of any that reports OOD rejection or risk-controlled abstention under a leakage-free protocol at a sub-1 KB budget.

### 2.2. OOD Detection in Medical Imaging

OOD detection for medical imaging is an active area surveyed broadly [10] with post hoc scores such as maximum softmax probability [11], the energy score [12], Mahalanobis [13], and k-nearest-neighbor OOD (KNN-OOD) [14] dominating practice. Recent medical benchmarks address vision-language OOD with semantic and covariate shifts [15] and computational-pathology OOD on whole-slide-scale models [16]. Open benchmarks also frame OOD detection as a deployment-safety and risk-control concern for medical classification [17]. These operate on server-scale models without an on-device budget, on histopathology or vision-language inputs rather than portable Giemsa microscopy, and without a leakage-free slide-disjoint protocol. CodaMal [18] performs contrastive domain adaptation between high- and low-cost malaria microscopes, but it adapts rather than abstains and is not sub-1 KB. Continual learning for malaria [19] spans multiple sites, but it is continual learning—not an abstention benchmark. A white-blood-cell domain-shift benchmark [20] establishes the microscopy-domain-shift benchmark genre, but it is single-pathogen and unconstrained in size.

### 2.3. Distribution-Free Risk Control

Conformal prediction [21,22], risk-controlling prediction sets [23], Learn-then-Test for selecting risk-controlling configurations [24], conformal risk control [25], and selective conformal risk control [26] give finite-sample distribution-free guarantees under exchangeable calibration samples. Conformal abstention has been applied to language and vision-language models [15]. When exchangeability fails, weighted and cluster-robust conformal variants [27,28] adjust the calibration estimator. We carry these guarantees into a regime where they had not been tested. We demonstrate on a public patch-clustered medical-microscopy benchmark that the naive cell-level certificate holds on only 20% of descriptive leakage-free re-splits. We also quantify what calibration at the slide unit costs in tightness, which is currently 0.266 at the slide count these public datasets supply. This empirical decomposition has not been reported for pathogen microscopy or for sub-1 KB integer on-device deployment.

### 2.4. Data Leakage in Medical-Imaging Benchmarks

Per-patch or per-cell random splitting that places samples from one patient or slide into both training and test inflates reported metrics, which is a recurring evaluation pitfall. The NIH malaria dataset is a canonical case [2], with roughly 200 patient slides, yet the near-universal protocol is a per-cell random split. We adopt slide-disjoint splitting throughout and show the same clustering that inflates per-cell accuracy also breaks the independence assumption of distribution-free certification, unifying the evaluation-rigor and certification-rigor arguments under one cause.

In summary, compact pathogen detectors, medical OOD detection, distribution-free risk control, and the data-leakage literature each address parts of the problem. To our knowledge, none couples a leakage-free slide-disjoint split, multi-pathogen microscopy OOD, a sub-1 KB integer on-device budget, and finite-sample risk-controlled abstention. None that we have found identifies that slide clustering breaks the distribution-free certificate. That four-way combination, together with the cluster-aware finding it makes visible, is what this paper contributes.

## 3. Method

**Figure 1:**
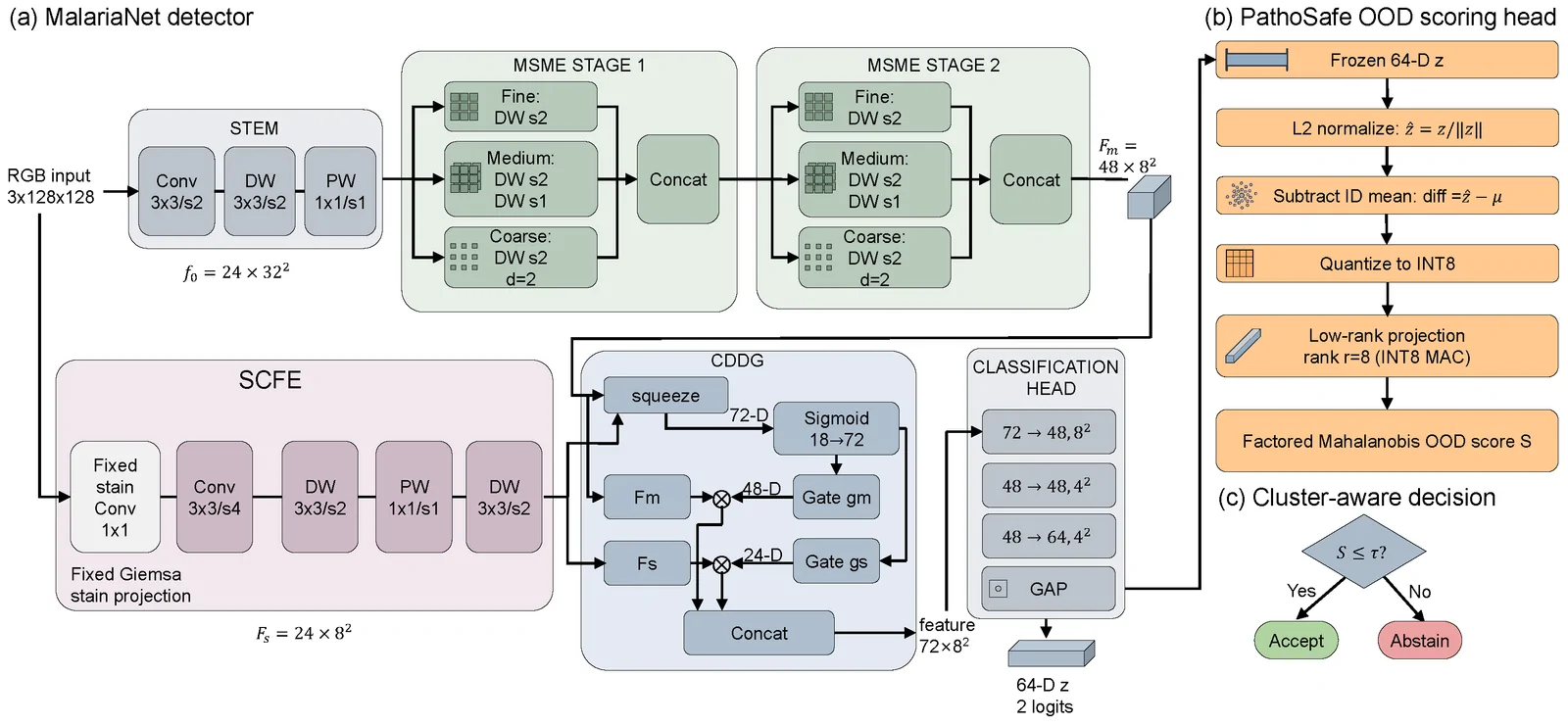
System overview. (**a**) The frozen 21 K-parameter MalariaNet detector, redrawn after [2]. The input feeds the stain-color feature extractor (SCFE) stream directly and the multi-scale morphology encoder (MSME) stream through the Stem, and the cross-domain diagnostic gate (CDDG) fuses the resulting maps $F_{s}$ and $F_{m}$ through the gates $g_{s}$ and $g_{m}$; the symbol ⊗ denotes element-wise gating (multiplication) of two feature maps. (**b**) PathoSafe scores the resulting 64-D penultimate feature *z*. (**c**) The score is compared with a cell-level calibrated threshold, producing accept or abstain.

We state the deployment setting and its operating assumptions (Section 3.1), then specify, in turn, the frozen backbone and feature locus (Section 3.2), the leakage-free slide-disjoint protocol (Section 3.3) and the reference OOD scoring system (Section 3.4). We then give the leaderboard baselines (Section 3.5) and how we calibrate the abstention threshold and correct its certificate to the slide unit (Section 3.6). Section 3.7 puts the head on the chip. Each is specified in the detail needed to reproduce it. Figure 1 summarizes the system.

### 3.1. Deployment Setting and Operating Assumptions

The constraints in this paper follow from one deployment picture. We state it here so the scope of each later result is explicit.

The device is a microcontroller-class portable microscope, a 400 MHz Cortex-M7 with a commodity camera in the sub-USD-60 class, running offline at a community health post. Flash and SRAM set the budget rather than compute, which is why the target is a sub-1 KB add-on. The detector is frozen at manufacture; in addition, no labeled data, no gradient path and no operator expertise for retraining exist on site, so the trustworthy layer must be post hoc. It reads the detector’s feature and changes neither its weights nor its decision. This also excludes scores that need input-gradient backpropagation.

Calibration happens before deployment. The abstention threshold is one of the 816 ROM bytes, which is fixed at build time from a held-out in-distribution calibration partition whose slide identities are known. The certificate is therefore a release-time artifact, which is why the budget binding it is counted in calibration slides rather than in anything the device meets later.

The unit of input is a single pre-segmented cell. Acquisition, autofocus, segmentation and whole-slide workflow sit upstream and are not addressed here. The output is accept or abstain on that cell. A decision layer above the head aggregates these into a slide- or patient-level action, for example referring a slide to expert microscopy once its abstention rate exceeds a threshold, and that layer is out of scope. The role is screening support under expert oversight rather than autonomous diagnosis, and the failure the head exists to remove is the silent one.

Two kinds of shift are expected in the field, and the OOD regimes of Section 3.3 stand in for them. The site, scanner or *Plasmodium* species may change while the question stays the same, or material outside the trained label space may reach the objective altogether.

### 3.2. Frozen Backbone and Feature Locus

The backbone is the fixed 21,085-parameter MalariaNet detector [2], a stain-morphology-decoupled malaria-cell CNN reused without modification and frozen throughout, so its behavior is bit-identical across sites and seeds. Its architecture is specified in that work and is summarized in Figure 1a only to locate the feature the OOD head reads. The OOD score reads the frozen penultimate feature (dimension d=64), denoted *z*, taken before any per-site adapter, in keeping with the frozen-detector premise of Section 3.1. Because the trunk does not depend on the training seed, *z* is deterministic across the five backbone checkpoints, which differ only in the per-seed adapter and head. This determinism is accounted for in Section 4.8; it is not presented as variance-free robustness.

### 3.3. Leakage-Free Slide-Disjoint Protocol

The cells of the NIH malaria dataset derive from roughly 200 patient slides. The conventional per-cell random split places cells from one slide into both training and test. This leaks slide-level staining and illumination, inflating accuracy estimates and the per-cell risk certificate computed on them. The leaderboard AUROC turns out to be insensitive to it for the structural reason given in Section 4.5. The protocol partitions slides into disjoint groups so that no slide contributes cells to more than one of training, in-distribution calibration, and test. The in-distribution (ID) task is NIH malaria. Two OOD regimes are evaluated against ID. *Cross-site* changes both the site and the *Plasmodium* species, the ID task being *P. falciparum* and BBBC041 *P. vivax*. *Multi-source* spans source types outside the ID label space—SIPaKMeD cytology, parasitic egg, and white blood cells—which are not all different pathogens. In the standard OOD taxonomy [10], cross-site sits closest to a covariate shift, in that the parasitized-versus-uninfected label space is unchanged, although the imaging site, the scanner and the species all differ. Multi-source is a semantic shift, where the inputs belong to classes outside the ID label space, so the two regimes probe distinct failure axes. The five-seed convention varies the per-seed adapter and classification head while the frozen trunk and the OOD split are fixed. The held-out ID calibration partition is used only for the abstention certificate. The slide grouping is recoverable from the public filenames. Every NIH malaria cell image carries the identifier of its source slide as a leading C-number, and grouping on that identifier under a GroupShuffleSplit reproduces the partition, so the protocol needs no extra artifact.

### 3.4. Reference OOD Scoring System

The reference is a low-rank Mahalanobis score [13,29] on the $\ell_{2}$-normalized frozen feature with int8 projection and floating-point score assembly. Let $\hat{z}=z/\max\left({∥z∥}_{2},\;10^{-12}\right)$ and $\mathrm{diff}=\hat{z}-\mu$ with μ the ID-train mean.

The empirical covariance of the ID-train normalized features $\Sigma \in R^{d\times d}$ admits the eigendecomposition $\Sigma =U\Lambda U^{⊤}$, $\Lambda =\mathrm{diag}(\lambda_{1},\ldots ,\lambda_{d})$, $\lambda_{1}\geq \cdots \geq \lambda_{d}\geq 0$. We keep the top-*r* eigenvectors as the column-stacked matrix $V_{r}=U_{:,1:r}$ and approximate the inverse covariance by a low-rank-plus-residual form,

(1) $$\Sigma^{-1}\;\approx \;V_{r}\;\mathrm{diag}\left(\mathrm{inv}\_\mathrm{wr}\right)\;V_{r}^{⊤}\;+\;\mathrm{inv}\_\mathrm{res}\;\left(I-V_{r}V_{r}^{⊤}\right),$$

with $\mathrm{inv}\_{\mathrm{wr}}_{i}=1/\lambda_{i}$ for i=1,…,r and $\mathrm{inv}\_\mathrm{res}=1/{\bar{\lambda }}_{r+1:d}$, which is the inverse of the mean residual eigenvalue. Substituting Equation (1) into the Mahalanobis quadratic form and writing $\mathrm{proj}=V_{r}^{⊤}\mathrm{diff}$ for the projection onto the kept subspace yields the numerically stable factored score

(2) $$S\left(\hat{z}\right)=\sum_{i=1}^{r}{\mathrm{proj}}_{i}^{2}\;\mathrm{inv}\_{\mathrm{wr}}_{i}+\mathrm{inv}\_\mathrm{res}\;\max\;\left({∥\mathrm{diff}∥}^{2}-\sum_{i}{\mathrm{proj}}_{i}^{2},\;0\right).$$

The factored form avoids assembling $\Sigma^{-1}$ densely. Its residual term is a difference of two positive quantities, ${∥\mathrm{diff}∥}^{2}$ and $\sum_{i}{\mathrm{proj}}_{i}^{2}$, which are close whenever the feature lies near the kept subspace, so finite-precision arithmetic can drive it slightly negative. The max(·,0) clamps it non-negative and leaves the score unchanged elsewhere.

#### Deployable Integer Arithmetic

On device, we replace fp32 operations with a fixed-scale int8 pipeline calibrated once on ID-train. With ROM-fixed scales *s* (for diff) and $s_{V}$ (for $V_{r}$), the int8-quantized forms are

(3) $$q_{d}=\mathrm{clip}\;\left(\lfloor \mathrm{diff}/s\rceil ,\;[-127,127]\right),\;q_{V}=\mathrm{clip}\;\left(\left\lfloor V_{r}/s_{V}\right\rceil ,\;\left[-127,127\right]\right),$$

where ⌊·⌉ rounds to the nearest integer. The projection on the kept subspace is then realized as an int8×int8 multiply-accumulate into an int32-range accumulator and rescaled in fp64,

(4) $${\mathrm{acc}}_{i}=\sum_{j=1}^{64}q_{V,ji}q_{d,j},\;p_{i}={\mathrm{acc}}_{i}\;\left(s\cdot s_{V}\right).$$

**Figure 2:**
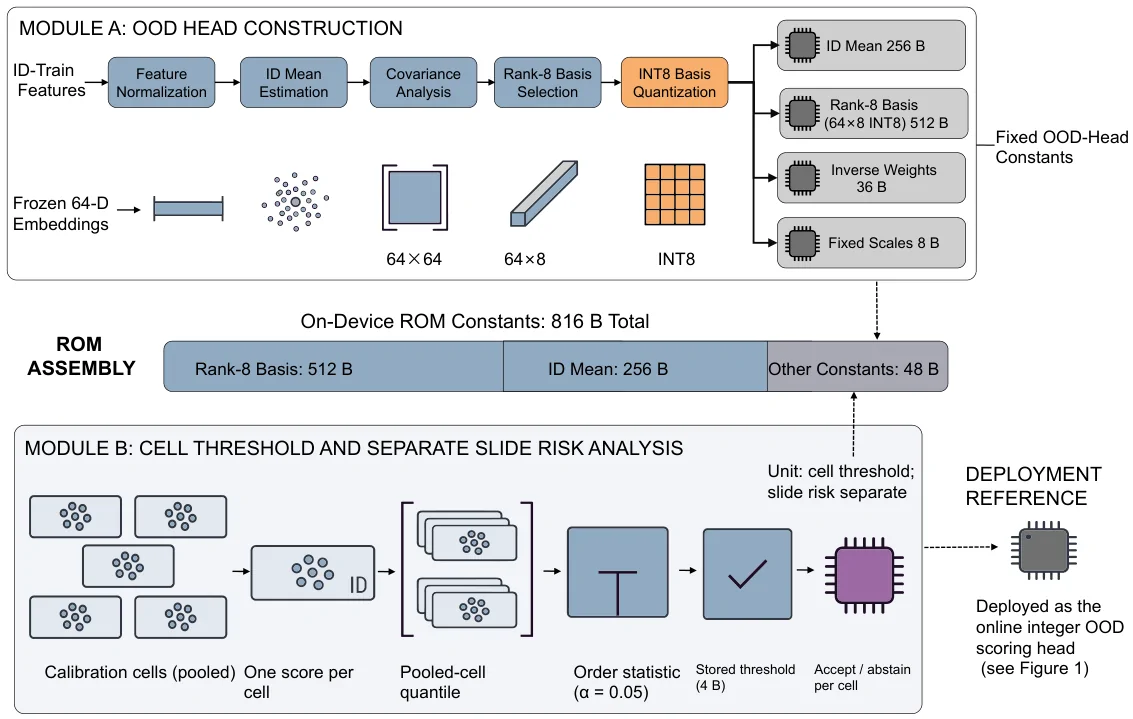
Construction of the fixed out-of-distribution (OOD) head constants and cell-level calibration. Module A: the in-distribution (ID) train features are normalized, their covariance is analyzed, and the rank-8 basis, ID mean, inverse weights, and fixed scales are selected and int8-quantized to assemble the fixed OOD-head constants (816 B total in read-only memory, ROM). Module B: cell-level calibration supplies the stored abstention threshold, and the separate offline slide-mean risk analysis is defined in Section 3.6; the latter does not set or certify the cell-level threshold.

With 64 terms and int8 codes bounded by 127, the largest absolute dot-product sum is 1,032,256, within the int32 range, so no explicit saturation is required. The deployed score reconstructs its total energy from the quantized codes,

(5) $$E_{q}=s^{2}\sum_{j=1}^{64}q_{d,j}^{2},\;S_{q}=\sum_{i=1}^{8}p_{i}^{2}\;\mathrm{inv}\_{\mathrm{wr}}_{i}+\mathrm{inv}\_\mathrm{res}\;\max\;\left(E_{q}-\sum_{i=1}^{8}p_{i}^{2},\;0\right).$$

which is the implemented approximation to Equation (2); the latter uses the unquantized difference and ideal basis. Figure 2 visualizes how the ID-train features construct the fixed head constants and how cell-level calibration supplies the abstention threshold. The corresponding online score path is shown in Figure 1.

On device, diff and $V_{r}$ are quantized to int8 using a single per-tensor scale calibrated once on ID-train. This scale is a ROM constant—not a per-batch runtime calibration over the data being scored. The on-device kernel is therefore deterministic across frames, and it matches the off-device reference within a measurable bound that Section 4.7 reports as $2.3\times 10^{-5}$ on embedded validation vectors. The projection is an int8 multiply-accumulate accumulated within the int32 range, and the score is assembled from the rescaled accumulators following Equation (5). The empirical advantage of per-tensor over per-group and per-channel quantization at this byte budget is shown in Section 4.4.

The stored ROM constants total 816 bytes. They comprise int8 $V_{r}$ codes (64×8=512 B), fp32 μ (256 B), fp32 inv_wr (32 B) and fp32 inv_res (4 B). The remainder is the fp32 feature scale and $V_{r}$ scale at 4 B each as well as the fp32 abstention threshold at 4 B. A higher score means more out of distribution.

### 3.5. Leaderboard Baselines

Under the identical protocol, on the per-seed adapted penultimate feature and over five seeds, we evaluate maximum softmax probability [11], the energy score [12], full-precision dense Mahalanobis [13] and KNN-OOD [14]. We add five post hoc comparators spanning the standard OpenOOD [30] families, namely ReAct [31], ASH-P [32], ODIN [33] at T=1000, ViM [34], and NNGuide [35]. ODIN stores no parameters but requires input-gradient backpropagation, which the deployed integer forward does not provide, so it is not microcontroller-deployable. For each, we report cross-site and multi-source AUROC and false-positive rate (FPR) at a 95% true-positive rate, as well as the on-device byte cost, including whether the method is sub-1 KB MCU-deployable at all.

### 3.6. ID-Scoped Risk-Controlled Abstention and the Cluster-Aware Correction

Abstention refers a low-confidence input to a specialist. We calibrate an abstention threshold by a distribution-free, finite-sample procedure. Given an ID calibration set of $n_{\mathrm{cal}}$ unit-level scores $\left\{S_{1},\ldots ,S_{n_{\mathrm{cal}}}\right\}$ and a target miscoverage α∈(0, 1), the split-conformal abstention threshold is the order statistic

(6) $$\hat{\tau }\;=\;S_{\left(k\right)},\;k=\left\lceil \left(n_{\mathrm{cal}}+1\right)\left(1-\alpha \right)\right\rceil ,\;\alpha =0.05,$$

where $S_{\left(k\right)}$ denotes the *k*-th smallest score. Under exchangeable calibration units, Equation (6) yields the marginal coverage guarantee $\mathrm{Pr}\left[S_{\mathrm{new}}\leq \hat{\tau }\right]\geq 1-\alpha$, i.e., the fraction of ID test inputs that pass the threshold is at least 1−α. This guarantee requires exchangeable calibration units; slide clustering violates this, so the cell-level coverage of $\hat{\tau }$ is reported as an empirical result rather than a formal guarantee, and the exchangeability-backed guarantee is the slide-level certificate introduced below. We use the selective form of [26] restricted to the in-distribution accept-region. Cross-site and multi-source inputs are non-exchangeable with the calibration set by construction, so no distribution-free coverage is claimed off-distribution and the OOD abstention rate at $\hat{\tau }$ is reported as descriptive.

#### The Certified Risk

The threshold $\hat{\tau }$ of Equation (6) controls *coverage*. The certificate controls a distinct *risk*. For a degraded in-distribution input (Section 4.2) with true label *y*, let $\ell =1[\hat{y}\neq y]$ be the per-cell degraded-misclassification loss of the deployed pipeline, front-end plus frozen detector. Its population degraded error is R=E[ℓ]. The certificate bounds *R* at confidence 1−δ by a finite-sample distribution-free Hoeffding–Bentkus upper bound [23]. It asks whether the best restoration front-end of the recoverability analysis (Section 4.2) drives *R* below the no-intervention floor. This degraded-error certificate is a separate guarantee from the abstention threshold $\hat{\tau }$, whose coverage form follows the same selective construction. The certificate itself involves no abstention and is evaluated on the full degraded stream. Coverage and risk are different objects but share one exchangeability requirement; hence, the single cluster-aware correction below applies to both. In this terminology, the cell-level coverage of Equation (6) concerns a random cell, and because cell-level exchangeability fails under slide clustering, it is empirical; the risk certificate concerns a cell from a random slide—equal-slide mean risk—with each slide contributing one exchangeable observation: the mean of its observed cells’ losses. We claim no whole-slide abstention guarantee.

The central methodological point is the unit of calibration. Treating the calibration cells as independent yields a tight-looking certificate that is anti-conservative under leakage-free re-splitting, because the effective sample size is governed by the number of slides rather than the number of cells. This follows from a cluster-sampling design-effect argument. With slides as clusters, the variance of any cell-averaged statistic is inflated by the design effect $\mathrm{DEFF}=1+(\bar{n}-1)\rho$, where $\bar{n}$ is the mean cells per slide and ρ the intra-slide correlation. The effective sample size is then $n_{\mathrm{eff}}=n/\left[\;1+\left(\bar{n}-1\right)\rho \;\right]$. Cells from one slide share staining and illumination, so ρ>0. We do not instantiate it numerically. The empirical re-split coverage (Table 1) settles the unit directly. The cell-level certificate holds on only 20% of leakage-free re-splits while the slide-level one holds on all, so the binding exchangeable unit is the ≈30 slides—not the ≈3600 cells. The slide is therefore the statistically correct unit of calibration, and that certificate is valid but conservative. Section 4.6 quantifies both and shows the naive cell-level certificate falls below the target in the descriptive re-split draws while the slide-level certificate holds. We expect this finding to extend to other patch-clustered medical benchmarks that apply a distribution-free guarantee at the patch level.

### 3.7. On-Chip Integer Deployment

The reference head is hand-coded as a mixed-precision int8 kernel. Its int8 quantizes the $\ell_{2}$-normed feature against the fixed scale and runs an int8 by int8 multiply-accumulate into an int32-range accumulator for the projections. It then assembles the factored score of Equation (5) from the dequantized accumulators and performs the threshold comparison. The target is an STM32H743 Cortex-M7 development board at 400 MHz. Latency is measured on that board with the data watchpoint and trace (DWT) cycle counter as the marginal cost added on top of the already characterized frozen-detector inference. The kernel’s score is validated against the off-device reference on embedded validation vectors so the deployed kernel is provably the evaluated algorithm. Section 4.7 reports the measured cycles, ROM and SRAM, and the numerical fidelity.

## 4. Experiments

This section reports the protocol and dataset setup; then, it reports the recoverability wall that closes off restoration as a research axis (Section 4.2) and the five-seed OOD leaderboard with per-source decomposition (Section 4.3). It then covers the integer-format ablation over rank and scale (Section 4.4) and the slide-split invariance of the leaderboard with its feature-versus-precision decomposition (Section 4.5). It then reaches the central result of the paper, the cluster-aware certificate diagnostic (Section 4.6), and closes with the on-chip STM32H743 measurement (Section 4.7) and the deployment caveats (Section 4.8).

### 4.1. Setup

The in-distribution task is NIH malaria [36] under the leakage-free slide-disjoint protocol of Section 3.3, in which no slide contributes cells to more than one partition. The recoverability wall (Section 4.2) and the certificate (Section 4.6) use this slide-disjoint partition. The OOD leaderboard is scored on a fixed in-distribution split, which Section 4.5 shows gives the same AUROC within 0.002 as slide-disjoint re-splitting, so it is not a split-leakage artifact. The cross-site OOD set is BBBC041 [37] (different site and *Plasmodium* species). The multi-source OOD set is SIPaKMeD cytology [38], parasitic egg (Chula-ParasiteEgg-11) [39], and white blood cells [20], which are not all different pathogens. All leaderboard scores are computed on the per-seed adapted penultimate feature (d=64), except the 816-byte reference, which reads the frozen 21 K detector’s penultimate feature directly, the trunk feature. The feature source is noted per row in the caption of Table 2. Results are five-seed. The backbone checkpoint varies while the OOD split is fixed. We report AUROC and FPR at a 95% true-positive rate.

### 4.2. Abstention Rather than Restoration

Before benchmarking abstention, we test the obvious alternative, restoring the degraded input so the frozen detector recovers. Under the same leakage-free slide-disjoint protocol, the detector is frozen and only a 2–3 K-parameter front-end is trained on per-sample mixed clean and degraded input. Degraded inputs are produced by a per-sample stochastic imaging-interference operator graded over six severity levels, L0 clean to L5 extreme. It composes color jitter of strength up to 0.55 with additive Gaussian noise, whose per-pixel σ is drawn uniformly from a level-dependent range up to [20,70] in 8-bit units. It then applies Gaussian blur and a downsample–upsample resolution reduction with probability up to 1.0. The front-ends are trained at the moderate L3 level with color strength 0.35, noise σ∈[10,40], blur applied and resolution reduction with probability 0.85, which are mixed per-sample with clean images at a 50:50 Bernoulli clean-to-degraded ratio under the slide-disjoint protocol. Degraded accuracy is then evaluated with the same operator applied to the held-out test cells. No task-driven restoration strategy shows a consistent advantage over plain pixel reconstruction on degraded accuracy. Over three seeds, pixel reconstruction reaches 92.75±2.05%. The three task-driven objectives reach 92.97±1.45% for a logit objective, 92.78±2.11% for a multi-layer feature anchor, and 91.58±3.15% for a decision-margin objective. At three seeds, the four strategies show overlapping accuracy, and a five-seed extension agrees. The five-seed paired differences from pixel reconstruction are +0.25, −0.44 and −0.70 percentage points for the logit, feature-anchor and decision-margin objectives, which are of mixed sign and well inside the 1.2 to 2.5 percentage-point between-seed spread of the strategies themselves. An exact Wilcoxon signed-rank paired test on those pairs gives p=0.63, 0.31 and 0.63. The smallest two-sided *p* attainable is 0.0625 at n=5, and 0.125 for the decision-margin pair, where one seed ties and leaves n=4, so the test cannot reject at any conventional level. We read it as a consistency check rather than as evidence of equivalence. As effect sizes, the paired mean differences relative to pixel reconstruction are +0.25 percentage points for the logit objective (95% interval, −0.93 to 1.44), −0.44 for feature anchoring (−1.43 to 0.54), and −0.70 for the margin objective (−2.61 to 1.21); these intervals include both no difference and potentially relevant changes. The five-seed degraded accuracies stay within about one percentage point of the three-seed means. The recoverability table and the certificate of Section 4.6 use these three-seed means with the five-seed result as a robustness confirmation. The no-front-end identity is 76.19±10.55%. Confidence-based quality gating, which abstains on the frozen detector’s own softmax confidence rather than on a distribution-aware score, does not reach a trustworthy operating point at usable coverage either. Over the same three seeds, the retained degraded accuracy at 75% coverage is 90.5%, 89.1% and 66.1%, and at 50% coverage, it is 95.3%, 93.2% and 70.6%. No seed approaches a trustworthy operating point. The spread tracks the degradation draw: the weakest seed is ungated at 60.9% against 83.3% and 82.8%, and it is the same seed behind the wide identity spread of Table 3. This pass applies the degradation operator under its own fixed seed, so its ungated 83.3% for seed 42 differs slightly from the 84.4% that seed contributes to the identity row of Table 3. Confidence and entropy gating coincide exactly here, as they must on a two-logit head where the two rankings are monotone transforms of each other. The clean-test baselines, reported in Table 3 for reference, are 94.3% for pixel reconstruction, 94.1% for the logit and feature-anchor objectives, 91.9% for the decision-margin objective, and 95.7% for the no-front-end identity. The 1–4 pp clean-accuracy compression is small, so the failure to beat pixel reconstruction on degraded input is not explained by clean overfit. The restoration axis therefore shows no consistent advantage for any task-driven variant, and confidence gating does not reach a usable operating point. What remains is abstention driven by an explicit out-of-distribution score rather than by the detector’s own confidence, which motivates the benchmark below. The degraded-input stream built here is retained for a second purpose. It supplies a per-cell loss with explicit slide structure, and it is on that loss that Section 4.6 demonstrates the cluster-aware certificate.

**Figure 3:**
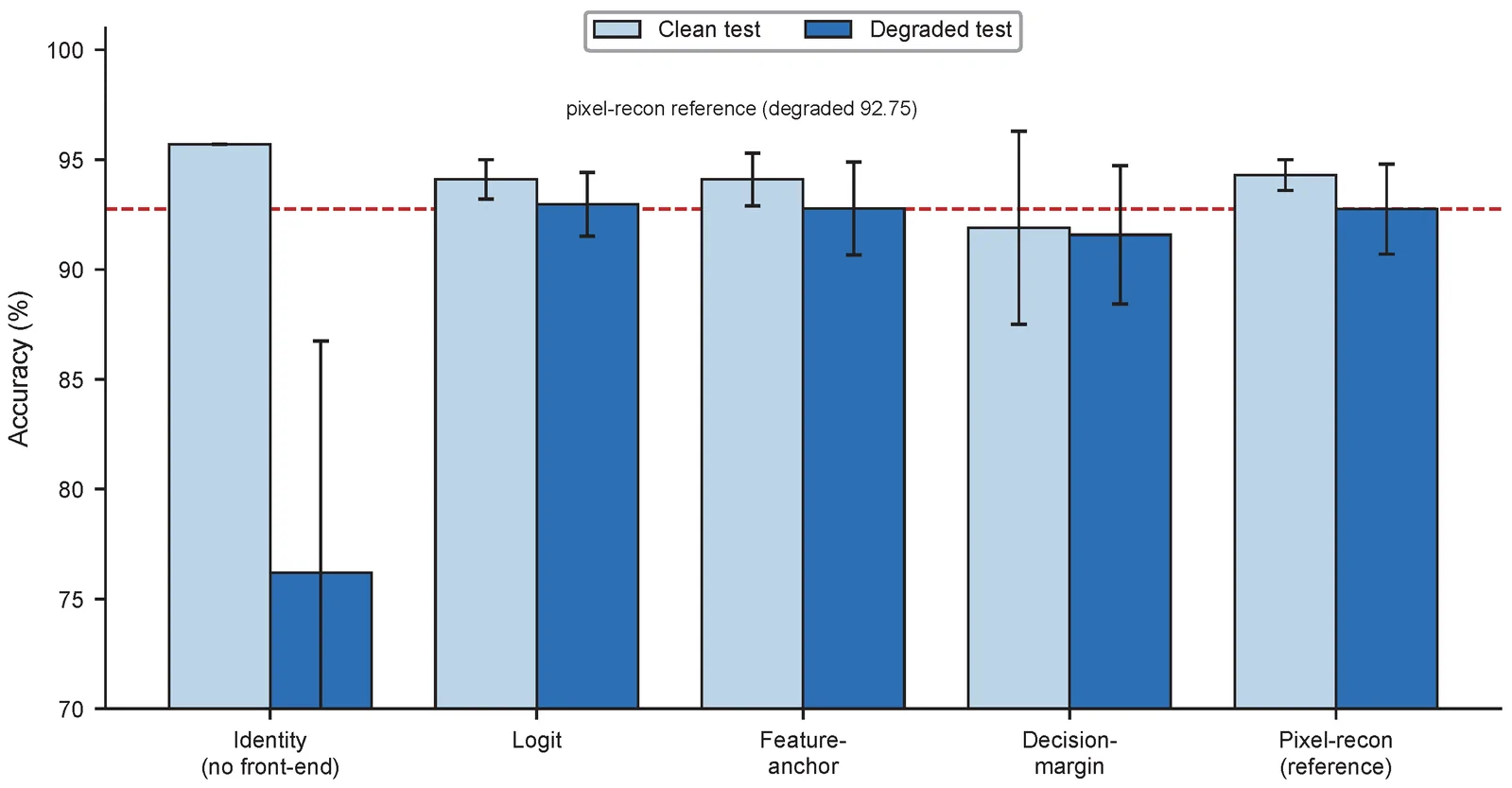
Recoverability wall, three-seed mean ± std. Clean and degraded test accuracy of the no-front-end identity, the three task-driven restoration strategies, and pixel reconstruction, with the pixel-reconstruction degraded reference marked by the red dashed line. Same data as Table 3.

We read this wall (Figure 3) as showing no consistent advantage rather than proven equivalence between the strategies. The pivot to abstention rests on the four objectives being indistinguishable from the simplest one rather than on ranking them: restoration itself recovers most of the degraded loss, which is why there is nothing further to win along that axis.

**Table 1:** Cluster-aware certificate diagnostic over 200 leakage-free slide-disjoint re-splits at the claimed coverage 1−δ=0.95. Wilson 95 % confidence intervals are computed from the binary pass/fail counts, and the binomial *P* tests $H_{0}$ that empirical coverage is at least 0.95. Risk bounds are the Hoeffding–Bentkus values on the held-out calibration split.

Calibration Unit	Cert. Risk	Pass Count	Emp. Coverage [95 % CI]	Binomial *P*	Interpretation
Cell (naive, iid)	0.054	40/200	0.20 [0.15, 0.26]	<$10^{-166}$	below target
Slide (correct)	0.252	200/200	1.00 [0.981, 1.000]	>0.95	above target

### 4.3. Leaderboard

Table 2 reports the five-seed leaderboard. The four post hoc scores that fit the sub-1 KB on-device budget, softmax, energy, ReAct, and ASH-P are all weak on cross-site at 0.775 to 0.812 AUROC. All four are computed from the two-logit detector head, whose binary output limits the expressiveness of post hoc confidence scores, so these values reflect this deployment form rather than a general failure of the methods. The stronger detectors are all outside the budget. Full-precision dense Mahalanobis is strong at 0.971 but costs 16,896 bytes. KNN-OOD, at 0.946, needs the full feature bank. ViM reaches 0.873 but stores an 8.4 KB residual subspace. NNGuide reaches 0.812 and, like KNN-OOD, needs the full feature bank. Input-perturbation ODIN adds no bytes but reaches only 0.801 cross-site, because its temperature scaling degenerates on a two-logit head, and it needs input-gradient backpropagation that the deployed integer forward does not provide. None of these five is sub-1 KB microcontroller-deployable.

**Table 2:** Five-seed leakage-free OOD leaderboard. Area under the receiver operating characteristic curve (AUROC) and false-positive rate (FPR) at a 95% true-positive rate (TPR) for cross-site (CS) and multi-source (MO); on-device bytes; microcontroller (MCU)-deployable (in the MCU column, y = deployable and n = not deployable). ^†^ The trunk-feature reference row is a single deterministic operating point rather than a five-seed mean; all other rows are five-seed. ^‡^ ODIN is not microcontroller-deployable (Section 3.5). Every row uses the per-seed adapted penultimate feature except the trunk-feature reference, which reads the frozen 21 K detector directly.

Method	CS AUROC	CS FPR95	MO AUROC	Bytes	MCU
Max softmax prob.	0.782	1.000	0.929	0	y
Energy	0.810	0.727	0.922	0	y
ReAct [31]	0.812	0.709	0.924	0	y
ASH-P [32]	0.775	0.781	0.924	0	y
ODIN [33]	0.801	0.774	0.931	0 ^‡^	n
Mahalanobis fp32 [13]	0.971	0.159	0.973	16,896	n
KNN-OOD [14]	0.946	0.179	0.971	bank	n
ViM [34]	0.873	0.561	0.947	8.4 K	n
NNGuide [35]	0.812	0.727	0.923	bank	n
Ours int8 (adapter feat.)	0.952	0.172	0.970	544	y
Ours int8 (trunk feat., ref.) ^†^	0.980	0.101	0.964	816	y

**Figure 4:**
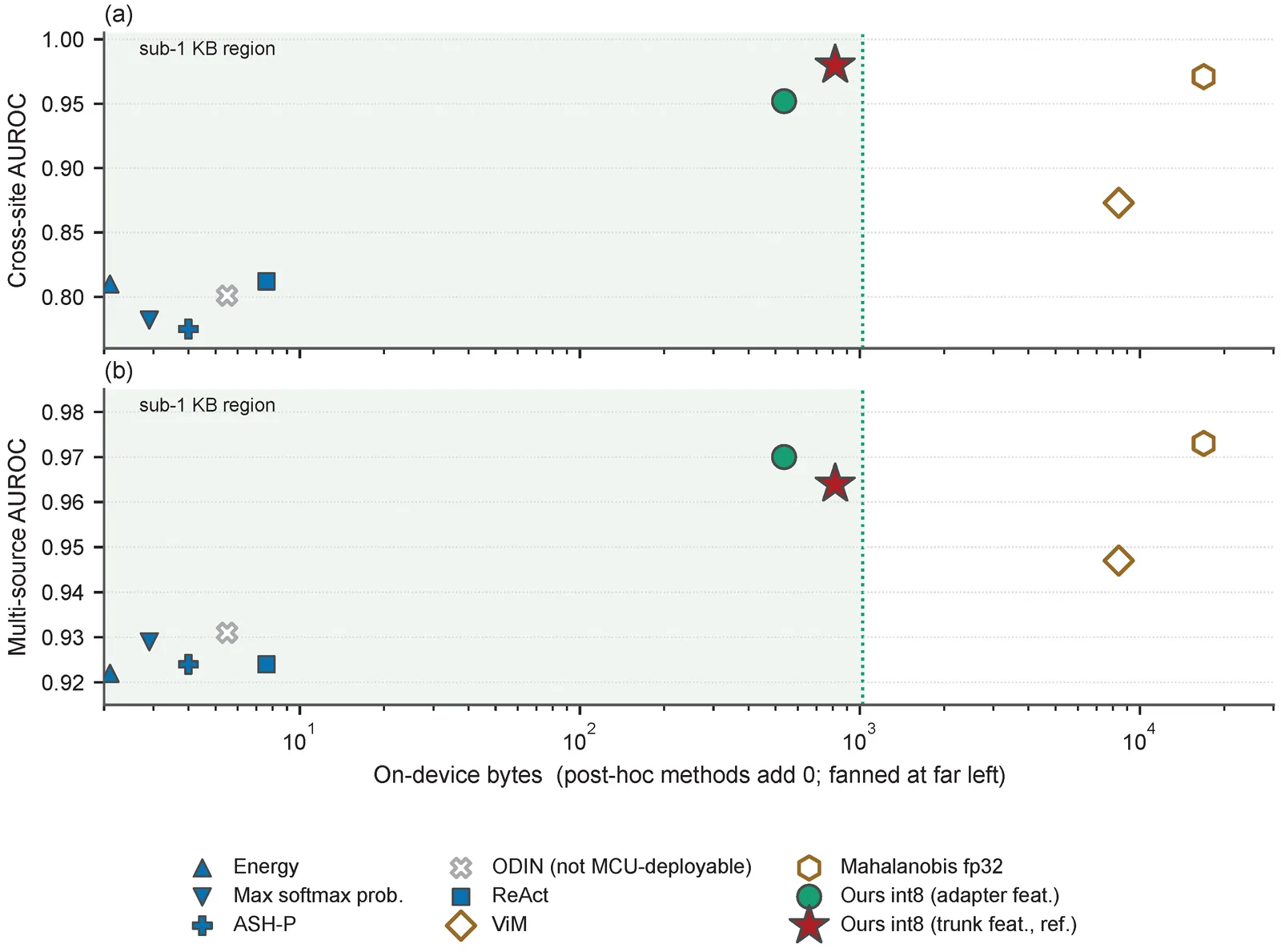
Byte-budget positioning of the leakage-free OOD leaderboard (Table 2) against on-device bytes for (**a**) cross-site and (**b**) multi-source. K-nearest-neighbor OOD (KNN-OOD) and NNGuide are omitted from both panels because a feature bank has no fixed byte cost; their values are in Table 2.

Figure 4 places the board on the byte axis. On cross-site, panel (a), our 816-byte int8-projection low-rank Mahalanobis reference, read from the frozen penultimate feature under deployable fixed-scale arithmetic, reaches 0.980. That is the highest of any microcontroller-deployable method and above the 16,896-byte native dense Mahalanobis at 0.971, while the four deployable post hoc scores top out at 0.812. The 544-byte adapter-feature variant reaches 0.952.

On multi-source, panel (b), the reference reaches 0.964 and the adapter variant 0.970. Among microcontroller-deployable methods, those are the two highest, against 0.929 for the best deployable baseline. Two methods outside the budget do edge ahead here, dense Mahalanobis at 0.973 and KNN-OOD at 0.971, so the ordering over the full board differs between the two regimes while the ordering within the budget does not. Relative to the adapter variant, the penultimate reference is a cross-site gain with a small multi-source trade-off, which is a different operating point rather than strict dominance.

The matched comparison describes what produces the cross-site advantage. On the same $\ell_{2}$-normalized trunk feature, the int8 head equals a full-precision dense Mahalanobis, 0.980 against 0.980 in Table 4, at about 21× less memory, and on the adapter feature, both sit near 0.95. The advantage is therefore associated with the feature source rather than the low-rank int8 form; the adapter is trained, so this comparison does not isolate feature location from training. On multi-source, the dense head on that same trunk feature is higher, 0.994 against 0.964, so the integer form trades multi-source separation for its byte budget rather than regularizing toward a gain. Note that this 0.994 and the 0.973 of Table 2 are not the same quantity. The latter is the Lee baseline under its own raw-feature convention, whereas Table 4 holds the feature fixed, which separates the feature-locus effect from the method effect (Section 4.5).

A test-set bootstrap with B=2000 resamples gives the reference 95% intervals: [0.978, 0.982] cross-site and [0.962, 0.967] multi-source. These quantify test-set sampling for a deterministic frozen feature, whereas the baseline scores are five-seed means quantifying training-seed variance, so the leaderboard mixes two uncertainty sources. Re-scoring every baseline at the reference seed and bootstrapping it identically removes that mismatch. On cross-site, all nine baseline intervals lie below the reference’s, the closest being full-precision dense Mahalanobis at [0.970, 0.975] against a reference lower bound of 0.978. On multi-source, dense Mahalanobis instead sits above it at [0.982, 0.986], which is the same trade-off Table 2 shows. Single-seed baseline estimates sit on either side of their five-seed means, so this check addresses the uncertainty comparison rather than the leaderboard ranking.

Decomposing the multi-source pool by pathogen (Table 5), the integer reference reaches AUROC 0.983 on SIPaKMeD cytology, 0.960 on parasitic egg, and 0.996 on white blood cells. The parasitic-egg subset dominates the pool by sample size and is the hardest source, because eggs share Giemsa-like staining and morphology with intra-cellular parasites. SIPaKMeD cytology at 0.983 and white blood cells at 0.996 are by contrast comparatively easy semantic shifts that separate cleanly. The genuinely demanding regimes are the cross-site covariate shift and this parasitic-egg near-OOD. The multi-source 0.964 aggregate is therefore set mainly by parasitic egg, 85% of the pool, not by the easier sources, and should be read as such rather than as uniform multi-pathogen difficulty.

**Table 3:** Recoverability wall (slide-disjoint, frozen detector, three-seed mean ± std; the certificate of Section 4.6 is derived from these three-seed means). No task-driven restoration strategy shows a consistent advantage over plain pixel reconstruction on degraded input. The five-seed paired differences are of mixed sign and well inside the between-seed spread with five-seed degraded accuracies within about 1 pp of these three-seed means.

Strategy	Clean Test (%)	Degraded Test (%)
Identity (no front-end)	95.7	76.19±10.55
Logit-only	94.1±1.0	92.97±1.45
Feature-anchor	94.1±1.2	92.78±2.11
Decision-margin	91.9±4.4	91.58±3.15
Pixel-recon (reference)	94.3±0.7	92.75±2.05

**Figure 5:**
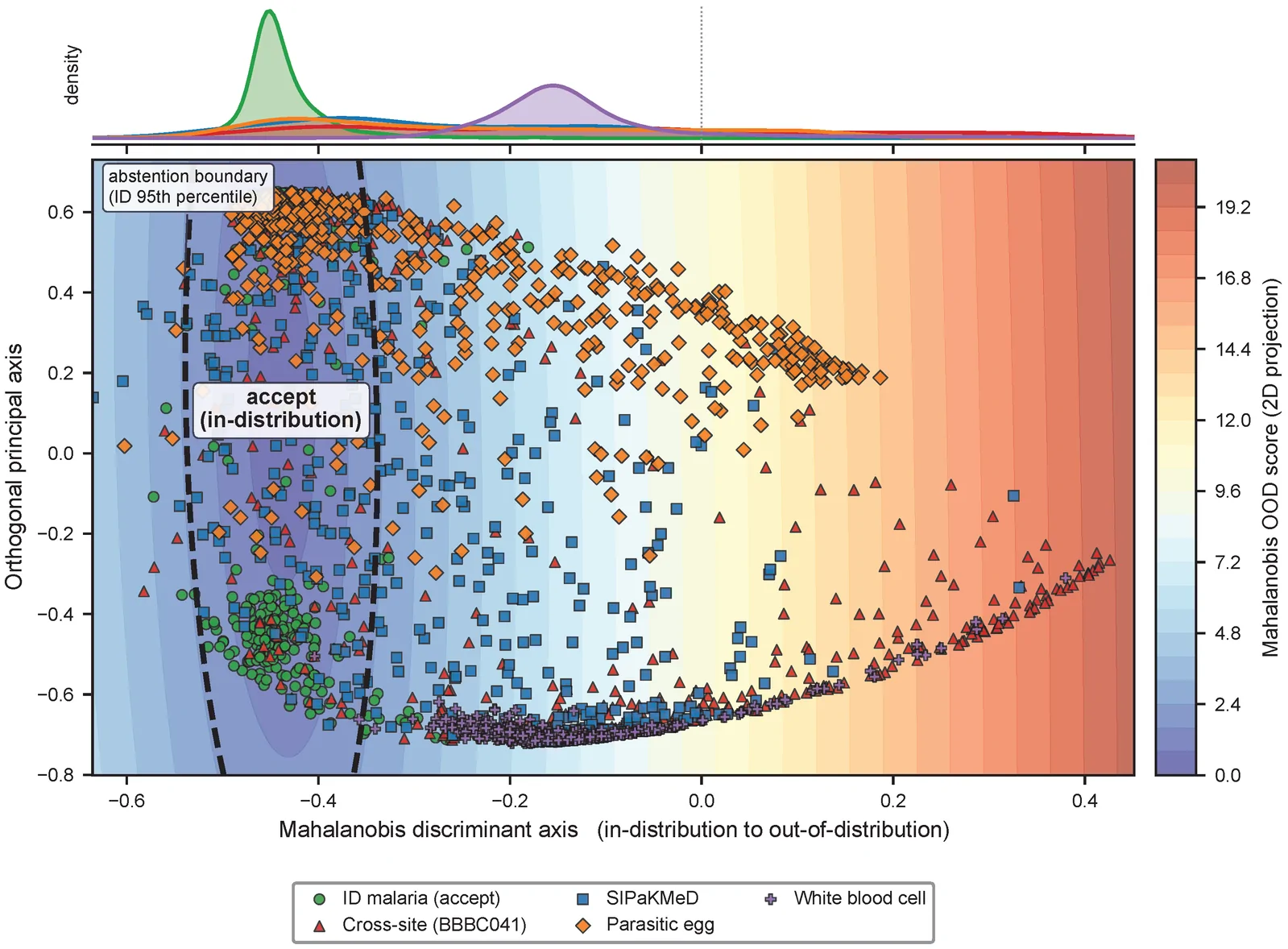
OOD-score landscape of the frozen-detector penultimate feature (d=64, $\ell_{2}$-normalized). The 64-D features are projected onto the Mahalanobis discriminant axis (in-distribution versus pooled out-of-distribution) and an orthogonal principal axis. The shaded field is the OOD score in this projection, the dashed contour an exploratory contour at the projected ID 95th percentile (distinct from the deployed threshold), and the top panel the per-source density along the discriminant axis. Plotted points are a 420-per-source subsample drawn for legibility.

**Figure 6:**
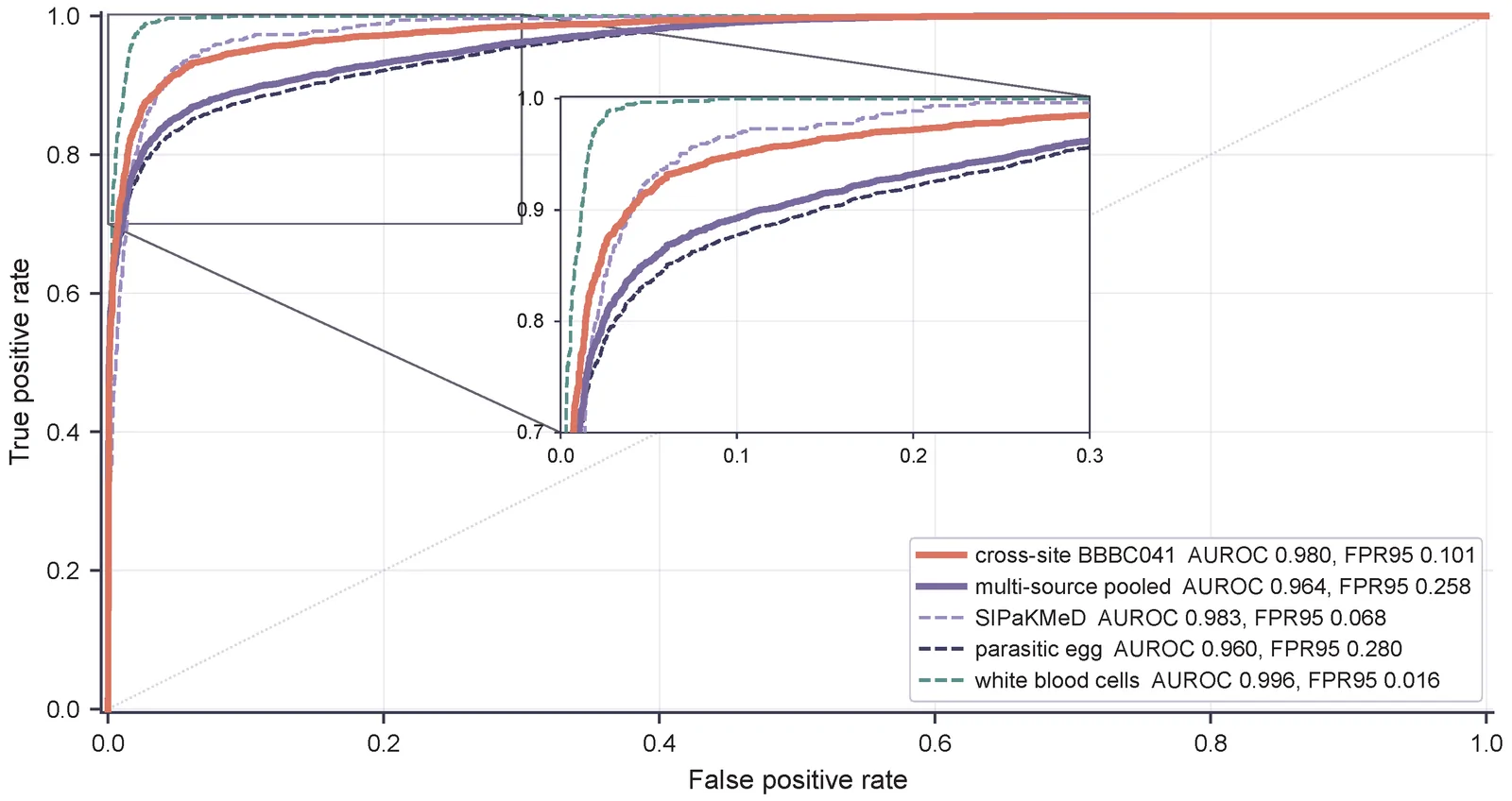
Receiver operating characteristic (ROC) curves of the 816-byte integer trunk-feature reference, ID test against each OOD set. The inset zooms on the operating region relevant for trustworthy abstention (false-positive rate ≤0.30, true-positive rate ≥0.70); the cross-site BBBC041 curve hugs the top-left corner with an FPR at 95% TPR of 0.101; white blood cells achieve 0.016, while parasitic egg is the hardest source at 0.280.

**Figure 7:**
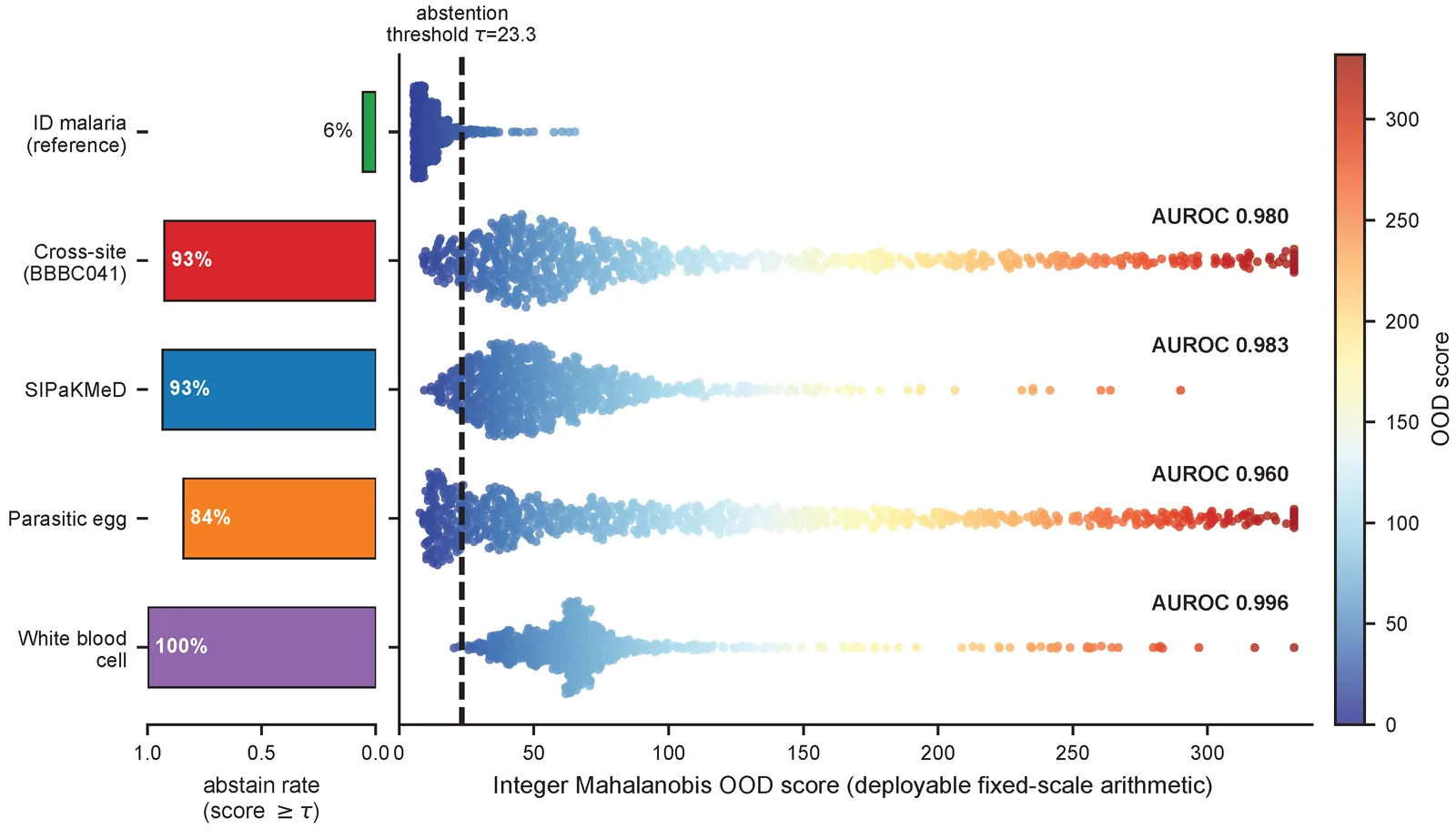
Per-OOD-source separation of the 816-byte integer trunk-feature reference. Each point is a scored cell (density-jittered swarm, ≈800 per source subsampled from all ≈21.9 K scored cells), colored by its integer Mahalanobis OOD score; the dashed line is the deployed ID-calibrated threshold $\hat{\tau }=23.27$. The left bars give the per-source abstention rate, the inline labels the ID-versus-source AUROC.

An OOD-score landscape of the frozen penultimate feature (Figure 5) makes this separation qualitatively visible. The in-distribution malaria cells form a dense cluster while the cross-site and multi-source OOD samples occupy distinct regions, which is the geometric structure the 816-byte integer head exploits. Figure 6 reports the ROC curves of the reference against each OOD set with the operating region around 95% TPR highlighted in the inset. Figure 7 then decomposes the score distributions by OOD source so the per-source overlap with ID test is directly visible.

Table 5 summarizes the per-source operating breakdown. The cross-site headline 0.980 and multi-source 0.964 are the two deployment-relevant aggregates. The per-source decomposition shows that the multi-source aggregate is bounded by the parasitic-egg subset. Parasitic egg is the hardest source, with route rate 0.843 versus 0.997 for white blood cells. It is also the dominant pool contributor by sample size at 85% of the pooled OOD test cells. At the deployed ID-calibrated threshold, the in-distribution false-abstention rate is 5.7% (235 of 4134 cells). About one in eighteen in-distribution malaria cells scores above $\hat{\tau }$ and is routed to expert review. For screening, this is the cost of the safety gate, which is a modest over-referral of in-distribution malaria-dataset cells in exchange for catching out-of-distribution inputs.

The score-distribution statistics behind these AUROC numbers are summarized in Table 6. The ID test scores are concentrated near zero, with median 9.7 and p95 24.4, while every OOD source has a median that exceeds the ID p95. Parasitic egg is the most shifted at the center, at median 84.9, and has the heaviest right tail, at p99 343.8, yet it also retains the largest fraction of cells below the threshold. That residual overlap, not a lack of shift, is why its AUROC is the lowest at 0.960. SIPaKMeD sits closest to the in-distribution body at median 50.2.

**Table 4:** Feature×precision 2×2 (CS/MO AUROC) on $\ell_{2}$-normalized features, comparing feature source against precision and rank. Dense rows are full fp32 class-conditional Mahalanobis at the reference seed. Integer rows are the deployed configs of Table 2, the trunk row being seed-invariant and the adapter row its five-seed mean.

Configuration	CS AUROC	MO AUROC
trunk feat., fp32 dense	0.980	0.994
trunk feat., int8 low-rank	0.980	0.964
adapter feat., fp32 dense	0.956	0.986
adapter feat., int8 low-rank	0.952	0.970

### 4.4. Memory–AUROC Trade-Off and Integer-Format Ablation

**Figure 8:**
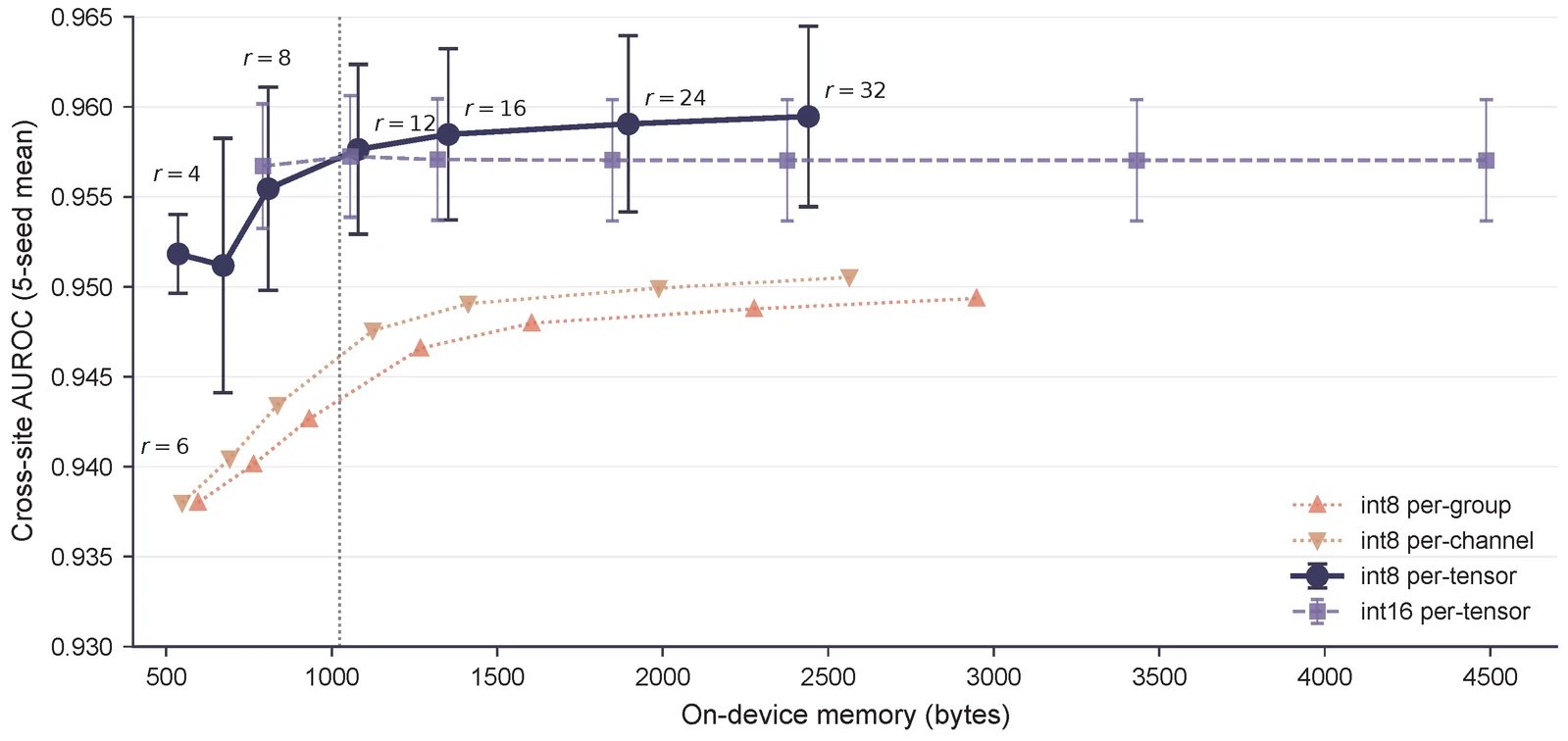
Cross-site AUROC versus on-device memory across rank, bit width, and scale granularity (five-seed mean, error bars are 1 std). The dotted vertical line marks the sub-1 KB on-device budget. This sweep is the adapter-feature head, which is a separate operating point from the 816-byte trunk-feature reference.

**Figure 9:**
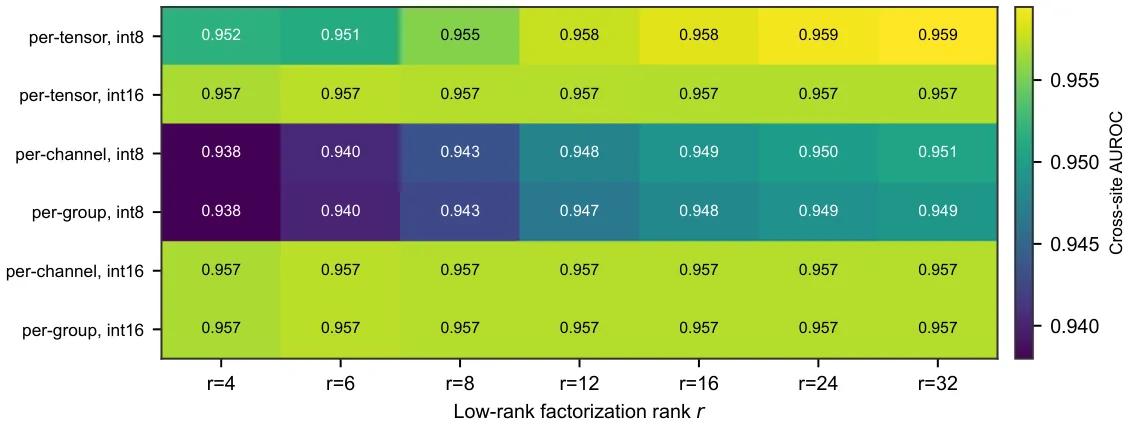
Cross-site AUROC across the full integer-format grid (rank r∈{4,…,32} by bit width and scale granularity, five-seed mean). int8 per-tensor is competitive with int16 and saturates by r=8, while int8 per-group and per-channel sit consistently lower at the same byte budget, supporting the deployed per-tensor int8 choice.

Table 7, Figure 8, and the full rank-by-scale heatmap (Figure 9) report a five-seed sweep over rank *r*, bit width, and integer-quantization scale granularity for the same factored low-rank Mahalanobis score. The leakage-free protocol and the frozen 21 K detector are held fixed. The adapter-feature path is used for this ablation because it varies with the training seed and supports a five-seed mean ± std. The trunk-feature reference is a single deterministic point of a frozen feature, which is reported separately in Table 2. Two effects matter for deployment. First, per-tensor quantization strictly dominates per-group and per-channel at int8 with r=4 int8 per-tensor reaching CS 0.952 against 0.938 for per-group and per-channel at comparable byte budget. This matches the deployable-fixed-scale analysis in Section 3.4. Second, cross-site AUROC saturates by r=8, the further 0.004 gain to r=32 sitting inside the five-seed spread of either row. Between r=4 and r=32, the int8 per-tensor family gains under 0.01 CS at 4.6× the memory, and at r=4 int16 buys 0.005 more CS than int8 but doubles the $V_{r}$ code size. The smallest configuration that both fits the sub-1 KB budget and holds a five-seed-mean CS AUROC of at least 0.95 is r=4, int8, per-tensor at 536 bytes of parameter subtotal (544 B with the feature scale and threshold). Its CS is 0.952±0.002 and its MO is 0.970±0.006, which is exactly the adapter-feature variant in Table 2. The deployed trunk-feature reference operates at r=8 and 816 bytes at the int8 per-tensor saturation point. Those 816 bytes are the 808-byte r=8 adapter-feature parameter subtotal plus 8 bytes of deployment-only constants (the 4-byte feature scale and the 4-byte threshold). A companion sweep over the same ranks on the trunk feature itself also peaks at r=8 on both axes and falls back slightly at r=16 and r=32. That sweep uses the per-batch scale rather than the deployed ROM-fixed scale, but it shows the deployed rank is not merely inherited from the adapter-feature ablation.

**Table 5:** Per-OOD-source operating breakdown of the 816-byte integer trunk-feature reference. AUROC and 95% bootstrap interval, FPR at 95% TPR, route rate at the deployed ID-calibrated abstention threshold ($\hat{\tau }=23.27$), and contribution to the multi-source pool by sample count.

OOD Source	*n*	AUROC [95% CI]	FPR95	Route@τ	Pool Share
BBBC041 cross-site	5452	0.980 [0.978, 0.982]	0.101	0.926	–
SIPaKMeD cytology	813	0.983 [0.979, 0.986]	0.068	0.935	7%
parasitic egg	10,532	0.960 [0.957, 0.963]	0.280	0.843	85%
white blood cells	975	0.996 [0.995, 0.997]	0.016	0.997	8%
multi-source pooled	12,320	0.964 [0.962, 0.967]	0.258	0.862	100%

**Table 6:** Score-distribution statistics of the 816-byte integer trunk-feature reference under the deployable fixed-scale arithmetic. All scores are computed from the same kernel that produces the leaderboard AUROC numbers (Section 4.3).

Class	*n*	Mean	Median	p95	p99	Max
ID test (NIH malaria)	4134	11.7	9.7	24.4	43.3	124.7
OOD BBBC041 cross-site	5452	103.5	69.3	287.3	328.9	364.4
OOD SIPaKMeD cytology	813	58.8	50.2	128.6	193.7	290.0
OOD parasitic egg	10,532	115.9	84.9	304.9	343.8	408.1
OOD white blood cells	975	73.4	65.3	159.7	263.1	332.1

### 4.5. Slide-Split Invariance of the Leaderboard

The leaderboard (Table 2) is scored on a fixed in-distribution split. To check that this is not a split-leakage artifact, we re-score the OOD AUROC over 200 leakage-free slide-disjoint re-splits of the in-distribution set, which is the same slide unit the certificate uses. Every method’s cross-site and multi-source AUROC stays within 0.002 of its fixed-split value with a per-split spread of at most 0.013 (Table 8). This invariance is largely structural. The OOD evaluation sets are separate datasets from the in-distribution malaria slides, so re-splitting the in-distribution set cannot introduce in-distribution-to-OOD slide leakage by construction. The re-split check confirms only that AUROC is also stable to the in-distribution split granularity, which it is because AUROC measures in-distribution-versus-OOD separation. The slide clustering that invalidates the cell-level certificate (Section 4.6) therefore does not inflate the leaderboard. Slide-disjoint splitting binds the certificate—not the leaderboard. The accuracy inflation this paper attributes to the same clustering is the detector’s own classification accuracy under a non-slide-disjoint split, which is a separate quantity from the OOD AUROC measured here. These values are at the reference seed, so the post hoc softmax and energy scores differ from the five-seed means of Table 2. The energy reads 0.699 versus 0.810 cross-site, which is a seed-variance offset that leaves the fixed-versus-re-split invariance intact. The wide cross-site energy spread across seeds is itself consistent with energy being, at the reference seed, the weakest cross-site score among the methods compared here.

**Table 7:** Five-seed integer-format ablation on the adapter-feature low-rank Mahalanobis score (rank *r*, bit width, scale granularity). CS and MO AUROC, mean ± std over five seeds; byte budget is a parameter subtotal (including μ) that excludes the 4-B feature scale and the 4-B threshold, so the full rank-4 and rank-8 payloads are 544 B and 816 B. Per-tensor strictly dominates per-group and per-channel at int8; cross-site saturates by r=8.

*r*	Bits	Scale	Bytes	CS AUROC	MO AUROC
4	8	per-tensor	536	0.952±0.002	0.970±0.006
6	8	per-tensor	672	0.951±0.007	0.972±0.002
8	8	per-tensor	808	0.955±0.006	0.973±0.002
12	8	per-tensor	1080	0.958±0.005	0.975±0.001
16	8	per-tensor	1352	0.958±0.005	0.975±0.001
24	8	per-tensor	1896	0.959±0.005	0.975±0.001
32	8	per-tensor	2440	0.959±0.005	0.975±0.001
4	8	per-channel	548	0.938±0.004	0.958±0.007
4	8	per-group	596	0.938±0.004	0.958±0.007
4	16	per-tensor	792	0.957±0.003	0.976±0.001

#### Feature-Versus-Precision Decomposition

To separate the feature-locus effect (trunk versus adapter) from the precision and rank effect (fp32 dense versus int8 low-rank), Table 4 fills the 2×2 grid at a fixed $\ell_{2}$ convention. The trunk feature reaches 0.980 cross-site under both the full fp32 dense Mahalanobis and the 816-byte integer head, while the adapter feature reaches only 0.952–0.956. The cross-site advantage is therefore associated with the feature source, not the precision or rank, although the adapter is trained and this comparison does not control for training. The dense rows are class-conditional while the integer head uses a single ID-train mean, so the grid also varies the conditioning structure. Because both still reach 0.980 on the trunk feature, neither precision, rank, nor conditioning is the driver, leaving the feature source as the remaining associated factor. Low-rank int8 quantization neither helps nor hurts cross-site on the trunk feature, and the deployed head matches full precision there at 21× less memory. On multi-source, the full dense detector is higher on both features, so the low-rank integer form trades multi-source separation for its byte budget. The fixed $\ell_{2}$ convention is itself not uniformly beneficial. On the adapter feature, it lowers cross-site (0.971 raw to 0.956) but raises multi-source (0.973 to 0.986), so the feature-locus effect should not be conflated with the $\ell_{2}$ normalization.

### 4.6. Measured Abstention Routing and Certificate Validity

**Figure 10:**
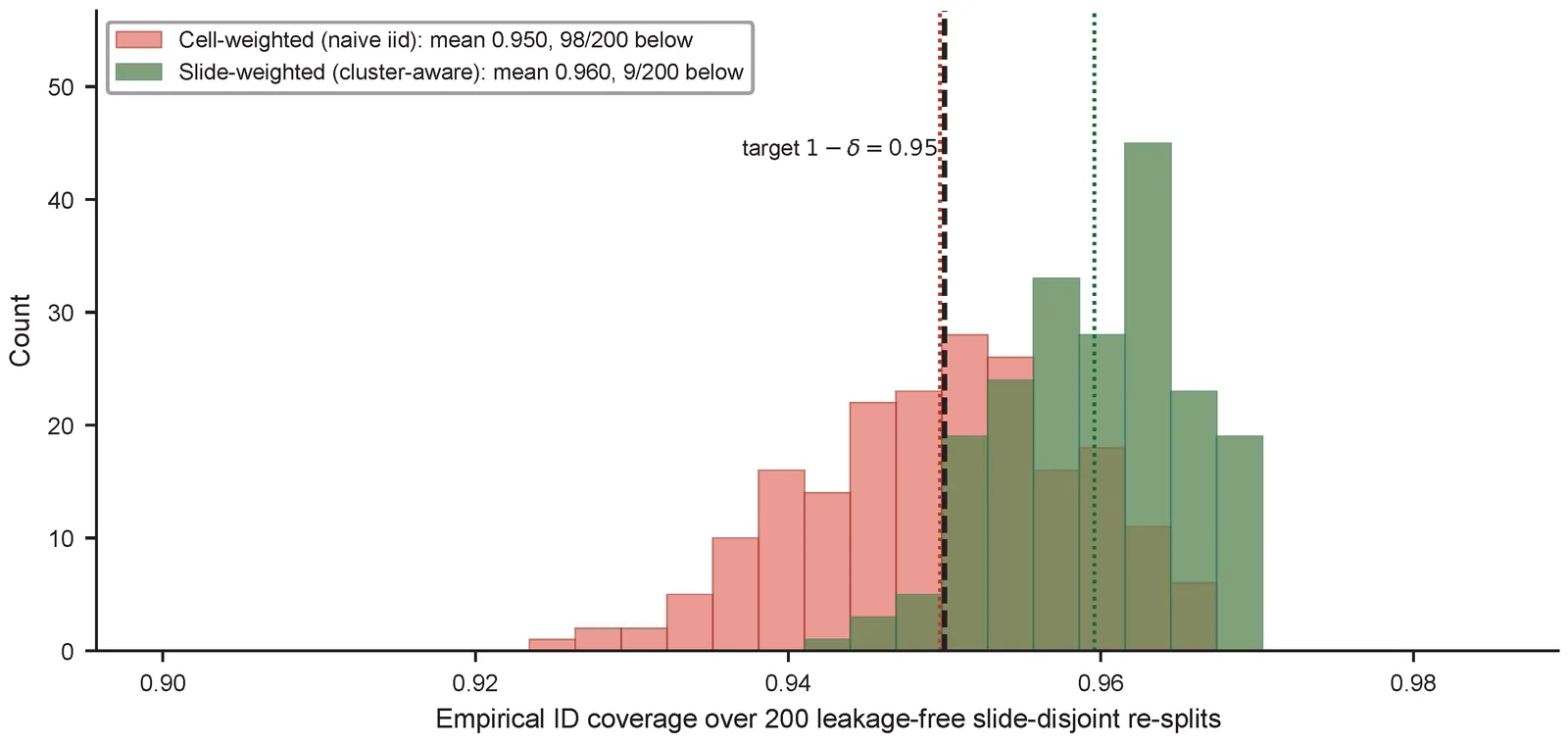
Distribution of the in-distribution coverage of the conformal abstention threshold $\hat{\tau }$ over 200 leakage-free slide-disjoint re-splits (the per-re-split values behind Table 9). The naive cell-weighted reading straddles the 0.95 target (mean 0.950, 98 of 200 re-splits below), while the cluster-aware slide-weighted reading concentrates above it (mean 0.960, 9 of 200 below); this is the same cell-versus-slide dependence as the degraded-error certificate, which is less severe but still present for the deployed threshold. The dashed vertical line marks the 0.95 target.

Abstention is calibrated by a distribution-free, finite-sample procedure scoped to the in-distribution accept-region (Section 3.6). Cross-site and multi-source inputs are non-exchangeable with calibration by construction, so off-distribution behavior is descriptive. At the ID-calibrated conformal-quantile threshold, the reference routes 92.6% of cross-site and 86.2% of multi-source inputs to a specialist. Among deployable methods, it is both the highest-AUROC score and the one with the lowest false-positive rate at 95% true-positive rate (0.101, Table 2), which is what makes a routing rate of this size attainable at an ID-calibrated threshold. Table 5 and Figure 7 report per-source routing at the ID-calibration split-conformal quantile $\hat{\tau }$ of Equation (6), which is the threshold actually stored among the ROM constants. The ID-test 95th percentile (τ=24.4) remains only a descriptive reference in Table 6. The deployed $\hat{\tau }$ carries the marginal ID-coverage of Equation (6), which is reported as an empirical result because cell-level exchangeability does not hold under slide clustering. Separately, the recoverability analysis’s degraded-error certificate (Section 4.2) is reported at the slide unit (Table 1), where it is valid. The cell-level risk certificate is anti-conservative and is *not* the one the deployed head relies on. Both guarantees are stated at the slide unit, so the high-routing operating point rests on the slide-level status rather than on the falsified cell-level one. We demonstrate the cell-versus-slide effect on the degraded-error certificate because it supplies a per-cell loss with explicit slide structure. It is the demonstration vehicle rather than the deployment guarantee. The abstention coverage shares the same exchangeable-calibration premise, so the same cell-versus-slide effect applies to it. An empirical re-split validation of the abstention coverage $\hat{\tau }$ itself (Table 9 and Figure 10) confirms this shared dependence. Over 200 leakage-free slide-disjoint re-splits, the naive cell-level reading meets the 0.95 target only on average (mean ID coverage 0.950) and falls below it on 98 of 200 re-splits. The cluster-aware slide-level reading is more conservative, at mean 0.960 and below target on 9 of 200, so the deployment guarantee inherits the cell-versus-slide dependence—though less severely than the degraded-error certificate. The mechanism is the split-conformal guarantee. The cell-based threshold is a 0.95 quantile, so its cell-weighted coverage centers on the target and dips below it on about half the re-splits. The slide-weighted reading gives each slide equal weight, is robust to large high-score clusters, and stays above 0.95 on all but 4.5%. Conditional coverage naturally varies across calibration sets, so the 98 of 200 cell-weighted readings below the target are not evidence that the empirical cell-level coverage is unreliable, and the 9 of 200 slide-weighted readings below it only indicate that the slide-weighted reading is more conservative. Meeting the target in mean therefore does not indicate not per-split reliability for the deployed threshold.

**Table 8:** Leaderboard AUROC is invariant to in-distribution split granularity. Fixed split versus 200 leakage-free slide-disjoint re-splits (mean ± std), reference seed. Both columns are that seed, so the softmax and energy values differ from the five-seed means of Table 2 by seed variance. Dense Mahalanobis here is $\ell_{2}$-normalized to match the integer head.

	CS AUROC			MO AUROC	
Method	Fixed	200 Re-Splits		Fixed	200 Re-Splits
Max softmax prob.	0.741	0.742±0.009		0.925	0.926±0.004
Energy	0.699	0.700±0.013		0.887	0.889±0.005
Mahalanobis fp32 ($\ell_{2}$)	0.956	0.956±0.005		0.986	0.986±0.002
Ours int8 (trunk feat., ref.)	0.980	0.981±0.003		0.964	0.964±0.006

**Figure 11:**
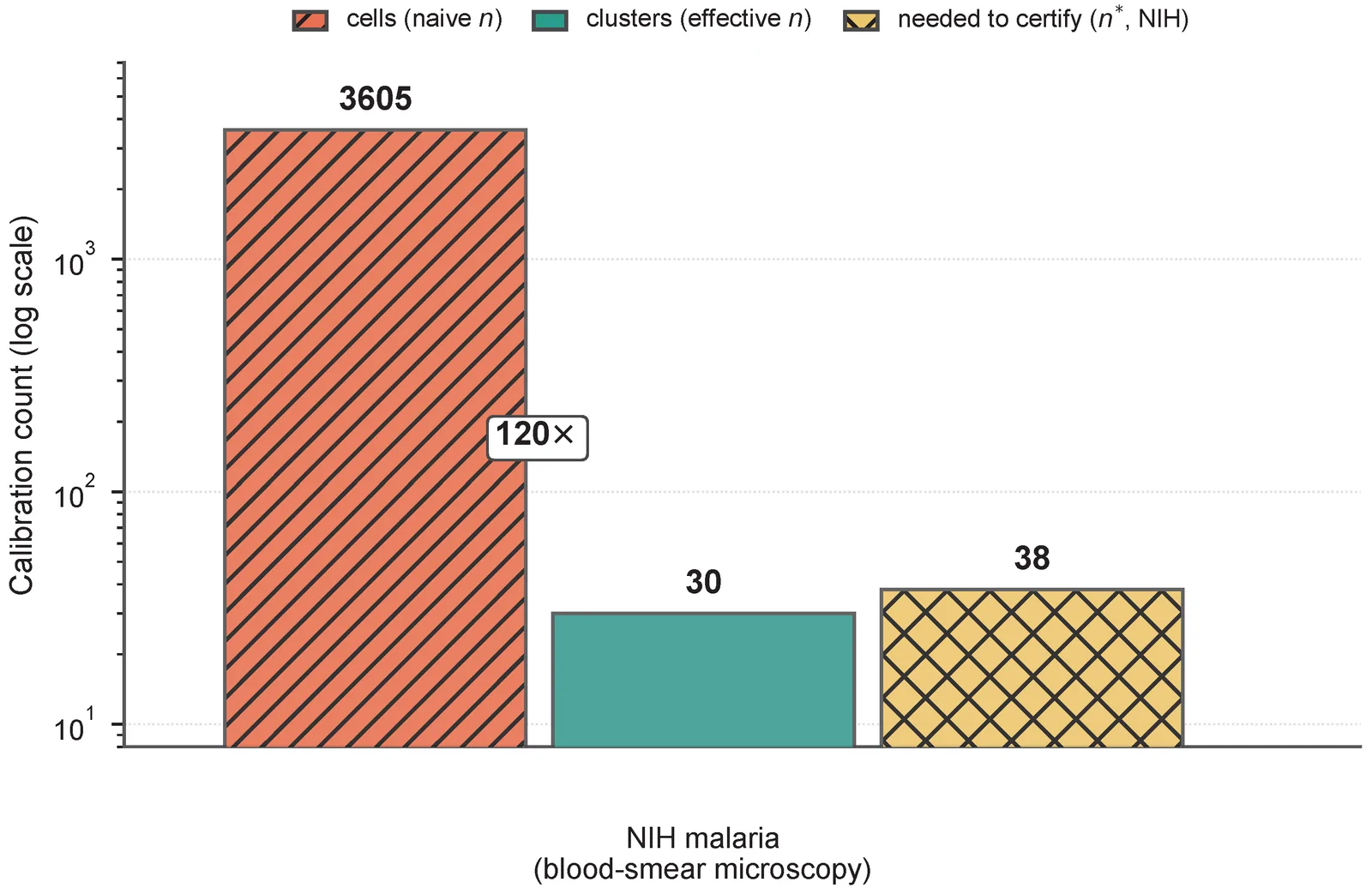
Naive versus effective calibration size on the NIH malaria benchmark. A naive certificate counts cells, but the exchangeable unit is the patient cluster: 3605 cells over 30 slides. Here $n^{*}$ denotes the number of slides required to certify improvement.

The central methodological finding concerns the calibration unit. Table 1 reports the cause-to-effect chain for the two calibration choices. The benchmark’s calibration partition is patch-clustered. The leakage-free slide-disjoint split leaves roughly 3600 calibration cells across only ≈30 patient slides. That is about a 120-fold gap between the cell count a naive certificate would use as *n* and the slide count that is the true exchangeable unit (Figure 11).

The naive cell-level certificate treats these ≈3600 cells as independent, producing a tight-looking risk bound of 0.054, yet it holds onto only 40 of 200 fixed-pool subset draws. The statistically correct slide-level certificate treats the 30 slides as the unit, gives a looser risk bound of 0.252 on the same calibration split, and stays above the target in all 200 draws. The chain is as follows. The per-cell loss *ℓ* is the degraded misclassification of Section 3.6 for the slide-best fixed front-end—not a per-slide oracle. That front-end is the feature-anchor variant, but it is not meaningfully different from plain pixel reconstruction on this benchmark (Section 4.2). Its per-slide mean over the 30 calibration slides, averaged over the three seeds, is the risk point estimate $\hat{R}=0.059$, which is the per-slide mean degraded error on the calibration partition. This differs from Table 3’s 92.78% degraded accuracy (error 0.072), which is the held-out test-set figure on a different partition. The per-seed finite-sample upper confidence bounds at n=30 slides average to 0.266. This 0.266 and the 0.252 above are the same slide-level Hoeffding–Bentkus certificate, 0.252 being its value on the single reference calibration split and 0.266 the conservative three-seed-mean carried as the headline figure. To be useful, it must fall below 0.239, which is the three-seed-mean degraded error of the raw input with no restoration and no abstention. That baseline is the *no-intervention floor.* Its held-out counterpart is the identity front-end of Table 3 at 76.19±10.55% degraded accuracy, the same quantity on a different partition, whose wide seed spread the floor inherits. It is the no-intervention floor by design—not a deployed restoration’s accept-all error. The latter would be lower and is not the bar a distribution-free release gate must clear. The bound does not fall below 0.239 at the current slide count, hence the slide-budget calculation below.

Read against that gate, the two calibration units return opposite verdicts on identical data. The naive cell-level bound of 0.054 sits far below the 0.239 floor and would appear to pass the gate, whereas the slide-level bound of 0.266 does not clear it. The cluster correction therefore does not merely widen the certificate; it reverses the decision the certificate exists to support, and the error it removes is in the permissive direction. The verdict on this benchmark is therefore that the intervention is not certified at the slides available, and the slide budget below is the price of a valid pass rather than a shortfall of the bound. A distribution-free abstention guarantee on this benchmark class should be computed at the slide unit and supplied with many more independent slides than a cell count suggests. A per-cell certificate should not be trusted on the strength of its width alone. Section 5 shows where this transfers and what slide budget it demands.

**Table 9:** Empirical ID coverage of the conformal abstention threshold $\hat{\tau }$ over 200 leakage-free slide-disjoint re-splits (target 1−δ=0.95; companion to Table 1). $\hat{\tau }$ is the 0.95 quantile of the calibration scores; coverage is read cell-weighted (every cell equal; naive ID) and slide-weighted (every slide equal; cluster-aware).

Reading	Mean ID Cov.	95% Range	Re-Splits < 0.95	Interpretation
Cell (naive iid)	0.950	[0.932, 0.965]	98/200	straddles target
Slide (cluster)	0.960	[0.948, 0.969]	9/200	conservative

**Figure 12:**
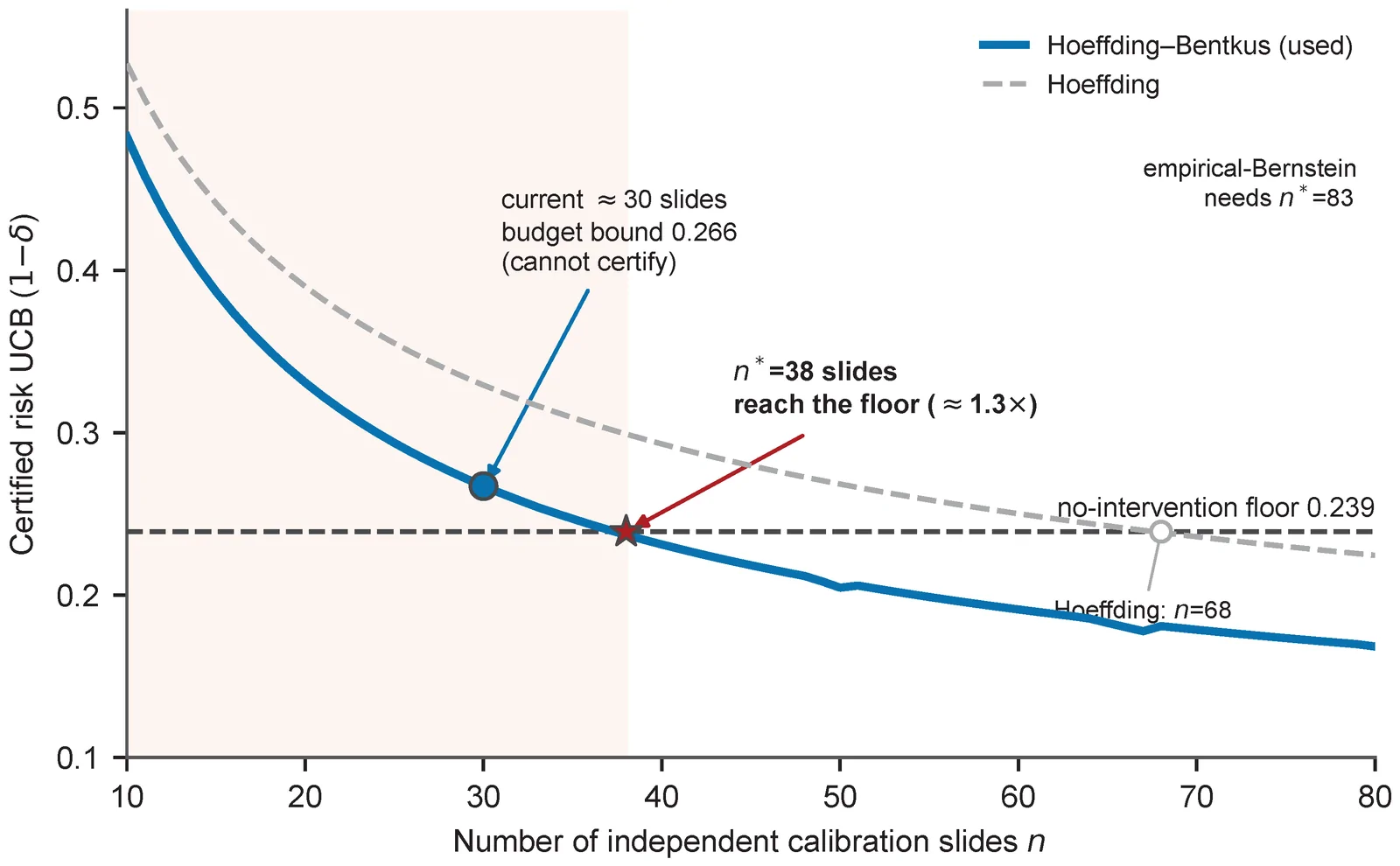
Cluster-aware certificate slide-budget curve. The finite-sample Hoeffding–Bentkus certified risk upper bound at per-slide degraded error $\hat{R}=0.059$ and δ/4 Bonferroni over the four-strategy family versus the number of independent calibration slides *n*. The curve is evaluated at the three-seed mean $\hat{R}$. The asymptotic slide bootstrap is omitted as it is not finite-sample valid at so few clusters. The horizontal dashed line marks the 0.239 no-intervention floor, and $n^{*}$ denotes the slide count at which the bound falls below it.

Cluster-robust calibrations do not rescue the certificate at the available slides. The slide-level certificate above is already cluster-aware. It aggregates per-cell losses to one risk per slide and applies the Hoeffding–Bentkus bound over the ≈ 30 slides. We additionally evaluated three further concentration bounds at the slide unit (Table 10, visualized in Figure 12). Among the finite-sample-valid bounds, the Hoeffding–Bentkus certificate is the tightest at 0.266. The plain Hoeffding at 0.329 and the variance-adaptive empirical-Bernstein bound [40] at 0.516 are looser at n=30 because their small-sample additive terms dominate. An asymptotic slide-cluster bootstrap appears tight at 0.098 but is not a finite-sample certificate at 30 clusters. It is the cluster-level analogue of the cell-level certificate that looks tight at 0.054 yet holds on only 20% of re-splits. Among the four bounds of Table 10, none certifies below the 0.239 no-intervention floor at the available slides. Bounds that exploit the unequal within-slide cell counts do not rescue it, either. The calibration slides hold between 65 and 315 cells, a coefficient of variation of 0.63, so weighting slides by their cell count lowers the risk estimate only from 0.050 to 0.044 while dropping the Kish effective sample size from 30 to 21.5. A size-weighted Hoeffding bound therefore widens to 0.364, and a two-level construction that bounds each slide from its own cells at δ/2n before combining across slides gives 0.473. Both are wider than the unweighted Hoeffding–Bentkus value, which is for the reason behind this paper: unequal cluster sizes cost effective sample size faster than weighting recovers it. On that evidence, the slide count, rather than the choice among the bounds tested, is what binds. The coverage-1.00 verdict is moreover established by re-splitting this same fixed pool of ≈ 30 slides, so it is internal validity for the available pool rather than external evidence for unseen slides or sites. Those re-splits are for the same reason not independent, so the binomial *P* of Table 1 is indicative rather than exact. The $n^{*}=38$ fresh-independent-slide requirement below is the other side of the same dependence. We solve the Hoeffding–Bentkus bound for the slide count that drives the (1−δ)=0.95 certificate below 0.239. The inputs are the three-seed-mean per-slide degraded error of 0.059 and the Bonferroni level δ/K=0.0125 over the K=4 restoration strategies. That gives $n^{*}=38$ independent slides, 68 under plain Hoeffding, or 83 under the empirical-Bernstein bound. This is of order a few tens and about 1.3× the post-disjoint NIH slide count. Recomputing the whole chain on all five seeds leaves the certificate at 0.266 and plain Hoeffding at 0.329 unchanged. The no-intervention floor rises to 0.250, the two added seeds including another hard degradation draw, and the requirement falls from 38 slides to 35. The three-seed convention is carried through the paper for consistency with Table 3; the five-seed recomputation is a robustness check rather than a competing headline. This $n^{*}$ is computed against the three-seed-mean no-intervention floor, which is itself a point estimate with a wide seed spread (Table 3, ±10.55) and no confidence bound. Putting a certified upper bound on one side of the comparison and a point estimate on the other is conservative for the intervention. It leaves the floor’s own uncertainty unaccounted, however, and a lower floor would demand more slides rather than fewer. $n^{*}$ is therefore an order-of-magnitude budget and a lower bound on the requirement rather than a precise count.

**Table 10:** Slide-unit concentration bounds (n=30 slides, Bonferroni δ/K, degraded-error risk). The 0.266 Hoeffding–Bentkus value is the three-seed-mean budget bound; on the single reference calibration split, it is 0.252 (Table 1).

Slide-Unit Bound	(1−δ) Risk	Finite-Sample Valid	<0.239?
Hoeffding–Bentkus [23] (used)	0.266	yes	no
Hoeffding	0.329	yes	no
Empirical-Bernstein [40]	0.516	yes	no
Slide bootstrap (asymptotic)	0.098	no (n=30)	appears yes

### 4.7. On-Chip Measurement

Table 11 reports the marginal cost of the integer reference head measured on an STM32H743 development board (Figure S1) at 400 MHz, added on top of the already-characterized frozen-detector inference, with the DWT cycle counter; Figure S2 shows the live serial-monitor capture of that run and the console log is reproduced in the Supplementary Materials. The head adds 12,304 cycles, 30.76 µs, with 816 bytes of ROM constants and 256 bytes of SRAM working memory; the matched compiled Flash increment is 1560 B for the scoring core and 1716 B with the normalization wrapper relative to a 17,492-byte control harness. The integer kernel reproduces the off-device reference on the embedded validation vectors to within $2.3\times 10^{-5}$ in score. The int8 multiply-accumulate is exact, and the residual is floating-point rounding in the fp64 dequantization and score assembly, which is returned as fp32. The threshold decision is correct for all validation vectors. The compact detector pipeline this head augments itself fits 23.5 KB int8 with a measured 816 ms scalar-C STM32H743 reference. The 30.76 µs abstention head is therefore a negligible fraction of the detector it protects. The 816 ms is an unoptimized scalar-C reference. A CMSIS-NN-optimized int8 detector kernel [41] would reduce it substantially, but across the plausible optimization range, the 30.76 µs abstention head remains a fraction of one percent of the detector cost. Section 5 reads these numbers for deployment.

### 4.8. Caveats

Two limitations are reported. The reference reads a training-seed-invariant frozen feature, so its cross-site AUROC is a single deterministic point. The uncertainty is therefore quantified by the B=2000 test-set bootstrap of Section 4.3 rather than being presented as zero-seed-variance robustness. The penultimate-feature reference is a cross-site gain with a small multi-source trade-off relative to the adapter-feature variant, 0.964 versus 0.970 multi-source, which indicates a different operating point rather than strict dominance.

## 5. Discussion

### 5.1. Why the Penultimate Feature Wins and What It Costs

The OOD score ceiling is set by the representation rather than the scoring head. Reading the frozen detector’s penultimate feature instead of an adapter feature changes the subspace the low-rank Mahalanobis score operates in, which is what lifts cross-site AUROC from 0.952 to 0.980 under the same int8-projection arithmetic (Section 4.3, Table 4). The gain is therefore associated with the feature source, not a quantization gain or a property of the scoring head, and it is not free. On the trunk feature, the rank-*r* truncation is lossless cross-site (0.980 for dense and low-rank alike) but costs multi-source AUROC (0.994 to 0.964). Truncation preserves the high-variance covariate-shift directions that dominate cross-site separation while discarding low-variance eigen-directions that carry semantic multi-source separation, which is consistent with the isotropic-residual inv_res approximation. The cross-site number is also a single deterministic point, because the frozen trunk does not depend on the training seed. We quantify its uncertainty by a B=2000 test-set bootstrap rather than as zero-seed-variance robustness. The 95% intervals are [0.978, 0.982] cross-site and [0.962, 0.967] multi-source. We present the adapter-feature variant alongside it so the trade is explicit rather than selected.

### 5.2. Deployment Significance

The integer reference head adds 30.76 µs, 816 bytes of ROM constants, and 256 bytes of SRAM on STM32H743 (Table 11), which is a fraction of one percent of the 816 ms detector it augments. This 816 ms detector latency is unrelated to the 816-byte head size. The integer kernel is numerically faithful to the off-device reference within $2.3\times 10^{-5}$. The OOD-rejection and abstention routing therefore add only a small measured cost on-chip. What is not yet free is the slide budget needed to certify them. On this axis, the obstacle to trusting a compact reader is not compute but evaluation and certification rigor, which is what this benchmark supplies. The upstream obstacles named in Section 3.1 remain separate problems that this paper does not address.

### 5.3. Slide Clustering and Distribution-Free Certification

The central methodological point is independent of malaria. A distribution-free certificate from conformal prediction, RCPS, or Learn-then-Test assumes exchangeable calibration samples. In compact-microscopy benchmarks, the natural sample is a cell, but cells are clustered within slides and are not independent. Treating them as independent yields a tight-looking certificate that holds onto only 20% of leakage-free re-splits. It is anti-conservative because the effective sample size is governed by the slide count rather than the cell count. The statistically correct slide-level certificate is valid, and at 0.266, it does not yet clear the 0.239 no-intervention floor it is meant to improve. What the correction changes is therefore the verdict rather than merely the width. The naive bound of 0.054 clears that floor and would appear to pass the gate, while the valid bound does not, so standard practice here returns a passing grade the data do not support. Its width is set by the number of calibration slides rather than by any estimator we substituted, so the quantity to report and to budget for is the cluster count. The cause is the same intra-slide non-independence that inflates per-cell accuracy under non-slide-disjoint splitting, so accuracy inflation and certificate anti-conservativeness share one cause. Any patch-clustered medical benchmark applying a distribution-free guarantee at the patch level is exposed to the same effect. It should report the effective cluster sample size and validate the certificate under cluster-disjoint re-splits. For a genuinely certified deployment, calibration must span many more independent slides than a cell-counted dataset suggests. Weighted and cluster-robust conformal variants [27,28] adjust the calibration estimator when the dependence structure is known and could in principle be applied at the slide unit, but we do not evaluate them here. What we do evaluate is the choice of concentration bound at that unit (Section 4.6, Table 10). At the available ≈30 slides, the Hoeffding–Bentkus certificate is already the tightest finite-sample-valid bound we evaluated, so within that family, the slide count rather than the choice of bound is what binds. Size-weighted and two-level variants are wider still, because the calibration slides differ enough in size that weighting costs a more effective sample size than it recovers. The $n^{*}=38$ independent slides derived there, about 1.3× what the NIH dataset provides post-disjoint, is the budget the field needs. Pathogen-microscopy datasets need to be expanded at the slide unit, not at the cell unit, if their distribution-free guarantees are to bind.

### 5.4. Broader Applicability of the Cluster-Aware Finding

The slide-clustering effect is not specific to malaria. We also attempted to check it on the BreakHis breast-histopathology archive [42], but that analysis could not be reconciled at the calibration-unit level: its summary records 41 calibration patients, whereas the original manuscript reported 20, and the per-image losses and split-generation records needed to resolve this discrepancy were not recovered. We therefore do not use the archived BreakHis summary as evidence of empirical confidence-bound validity or of a cross-modality transfer. Other patch-clustered medical imaging whose effective cluster count is far below its patch count plausibly shares the structure—retinal regions per fundus image, dermatology lesions per patient, volumetric CT and MRI slices per volume, and ultrasound frames per probe placement—but we have not measured these, and a distribution-free guarantee computed at the patch level on them should be validated at the cluster unit before it is trusted.

### 5.5. Clinical Significance and Scope

A potential use of the head is to flag cells for review when their feature scores exceed a stored threshold. The OOD score is not a diagnostic label or a general test of classifier confidence; erroneous predictions may still be accepted, and in-distribution cells may be rejected. Converting cell-level outputs into a clinical action requires a prespecified slide-level aggregation rule and evaluation on independent complete slides. The aggregation layer that turns per-cell decisions into a slide- or patient-level action, and the screening-support role it serves, are set out in Section 3.1. Validating that layer is left to a prospective field study.

### 5.6. Limitations

The OOD regimes are public proxies for site and species shift rather than a prospective field study. At the available slide count, the slide-level certificate is too wide to clear the no-intervention floor, and narrowing it requires more independent slides than current public datasets provide. The cross-site reference number is a single deterministic point, which is quantified by a B=2000 test-set bootstrap rather than seed variance. The benchmark is demonstrated on malaria-centered microscopy. The cluster-aware certification finding is general but is quantified here on one benchmark: malaria microscopy. The on-chip work is a development-board characterization of the model rather than a fielded instrument. It covers only the abstention head on pre-segmented cells.

The board experiment characterizes the scoring of stored feature vectors; it does not measure an integrated acquisition pipeline. Future camera integration on the STM32 controller must account for frame buffers, shared memory and bus traffic, DMA/cache coordination, storage or display load, and exposure, focus, and synchronization control. As a storage illustration, one 640×480 RGB888 frame contains 921,600 bytes before additional buffers or processing workspace; this is not a tested camera configuration. Camera readout, segmentation, backbone inference, end-to-end throughput, power, and battery life remain outside the present measurements.

The evaluated OOD sets cover heterogeneous acquisition sources and image morphologies. BBBC041 changes acquisition source and *Plasmodium* species together, so it does not isolate a single shift. The multi-source pool contains cytology, parasitic eggs, and white-blood-cell images; these are not all different pathogens, and eggs account for 10,532 of 12,320 pooled inputs (85.5%), making the aggregate result composition dependent. The present experiments do not establish robustness to clinically plausible near-distribution changes such as stain precipitate, overlapping cells, low parasitemia, or focus errors.

## 6. Conclusions

We presented three contributions for trustworthy on-microcontroller pathogen microscopy. The first and central one is a cluster-aware certification finding, which puts the calibration unit rather than the estimator at the center of any distribution-free guarantee issued on clustered medical data. The second is a leakage-free benchmark and protocol for out-of-distribution rejection and risk-controlled abstention. The third is a sub-1 KB int8-projection reference system that brings OOD scoring inside the on-device budget of a commodity microcontroller.

The central finding is general. Slide clustering silently breaks the standard independent-sample distribution-free certificate. On the degraded-input risk certificate, a naive cell-level version is anti-conservative, holding onto only 20% of leakage-free re-splits, while the statistically correct slide-level version is valid at 0.266. The deployed abstention threshold inherits the same dependence less severely. The width of a valid certificate is set by the number of slides rather than by any of the bounds we evaluated, which is what makes the finding actionable. A Hoeffding–Bentkus budget gives an illustrative count of 38 independent slides under the stated assumptions (35 when five seeds are used), which is about 1.3× what this benchmark provides. The same intra-slide non-independence also inflates per-cell accuracy, so two rigor failures share one cause. The effective sample size of a patch-clustered medical certificate is governed by the number of clusters rather than the number of patches. Slide-disjoint evaluation and slide-level, cluster-aware certification should both be standard for this benchmark class.

The reference system serves that agenda rather than competing on accuracy. On the leaderboard, it gives the strongest cross-site separation of any sub-1 KB microcontroller-deployable method at AUROC 0.980. Its advantage is a feature-source effect rather than a quantization gain. On an STM32H743 development board, it costs a measured 30.76 µs and 816 bytes of ROM constants, so abstention adds only a small measured cost on-chip. The recoverability wall, on which no task-driven front-end separates from plain pixel reconstruction, is what redirected this paper from restoration to benchmarking and certifying abstention.

Two caveats qualify these results: the deterministic single-point nature of the frozen-feature cross-site number and the trade-off between cross-site and multi-source AUROC. Cluster-robust conformal calibration over many independent slides and prospective field validation are the natural next steps toward genuinely certified point-of-care deployment.

## Figures and Tables

**Table 11:** Measured on-chip marginal cost of the integer reference head, STM32H743 development board at 400 MHz, data watchpoint and trace (DWT) cycle counter, averaged over 200 iterations. Latency, ROM and static random-access memory (SRAM) are measured on that board; the fidelity row is the maximum absolute score difference between the compiled integer kernel and the off-device reference over the embedded validation vectors. The measurement characterizes the model on that board and is not a field trial.

Quantity	Measured
Marginal latency	12,304 cyc/30.76µs
ROM constants	816 B
Compiled Flash increment	1716 B (1560 B core)
SRAM working	256 B
Kernel fidelity (max |C−ref|)	$2.3\times 10^{-5}$

## Data Availability

The in-distribution NIH Malaria Cell Images dataset [36] and the cross-site BBBC041 *P. vivax* dataset [37] are publicly available; the multi-source out-of-distribution sets, SIPaKMeD cervical cytology [38], the Chula-ParasiteEgg-11 parasitic-egg dataset [39], and a five-class white-blood-cell dataset [20] are likewise publicly available from their original providers. The leakage-free slide-disjoint split is reproducible directly from the public NIH filenames, using the GroupShuffleSplit on the leading C-number slide identifier specified in the Methods, so no additional artifact is required to reproduce the protocol or the certification analysis. The source code, the trained detector and integer reference-head constants, the integer kernel with its on-chip validation vectors, and the evaluation and certification scripts will be deposited in a public repository (Zenodo) upon acceptance, and these are available from the corresponding author in the interim.

## Supplementary Materials

The following supporting information can be downloaded at: https://www.mdpi.com/article/10.3390/bios16090518/s1, S1—Development-board photograph and live on-chip measurement; S2—On-chip measurement console log. Figure S1: Photograph of the STM32H743 development board (ATK-H743) on which the integer OOD head was measured and run. The board is powered on (status LEDs lit) and connected to the host over USB. The head firmware (head_with_normalization; low-rank *r* = 8 int8 projection with fp32 scale assembly, 816 B of ROM constants) runs on this board. Figure S2: Live serial-monitor capture (COM3 @ 115200 baud, 8N1) of the running firmware reporting the 400 MHz DWT measurement. The highlighted line reports avg_cycles = 12,304.415 over 200 calls (i.e., 30.76 us at 400 MHz) with max_abs_score_error = 2.29e-5 against the offdevice reference over the eight embedded validation vectors.

## Abbreviations

The following abbreviations are used in this paper:

OOD	Out-of-Distribution
ID	In-Distribution
CS	Cross-Site
MO	Multi-source (multi-pathogen) Out-of-distribution
ROC	Receiver Operating Characteristic
AUROC	Area Under the Receiver Operating Characteristic
FPR	False Positive Rate
TPR	True Positive Rate
MCU	Microcontroller Unit
ROM	Read-Only Memory
SRAM	Static Random-Access Memory
int8	8-bit Integer Quantization
fp32	32-bit Floating Point
RCPS	Risk-Controlling Prediction Set
KNN	K-Nearest Neighbors
NIH	National Institutes of Health
BBBC	Broad Bioimage Benchmark Collection
DWT	Data Watchpoint and Trace
CNN	Convolutional Neural Network
SCFE	Stain-Color Feature Extractor
MSME	Multi-Scale Morphology Encoder
CDDG	Cross-Domain Diagnostic Gate
