# Supplementary material for: PathoSafe: Sub-1 KB Out-of-Distribution Rejection and Cluster-Aware Certification for Point-of-Care Pathogen Biosensing
Source: Biosensors (Basel). 2026 Sep 17;16(9):518. doi: 10.3390/bios16090518 (PMC13604659; doi:10.3390/bios16090518)
Supplement: Supplementary file 1 [file biosensors-16-00518-s001.zip › biosensors-4534019-supplementary.pdf]

Supplementary Material

Manuscript: biosensors-4534019 (PathoSafe)

S1 — Development-board photograph and live on-chip measurement

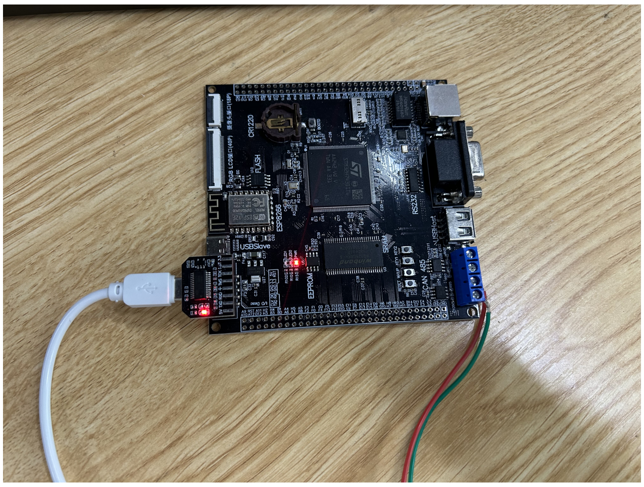

Figure S1: Photograph of the STM32H743 development board (ATK-H743) on which the integer OOD head was measured and run. The board is powered on (status LEDs lit) and connected to the host over USB. The head firmware (head_with_normalization; low-rank $r = 8$ int8 projection with fp32 scale assembly, 816 B of ROM constants) runs on this board.

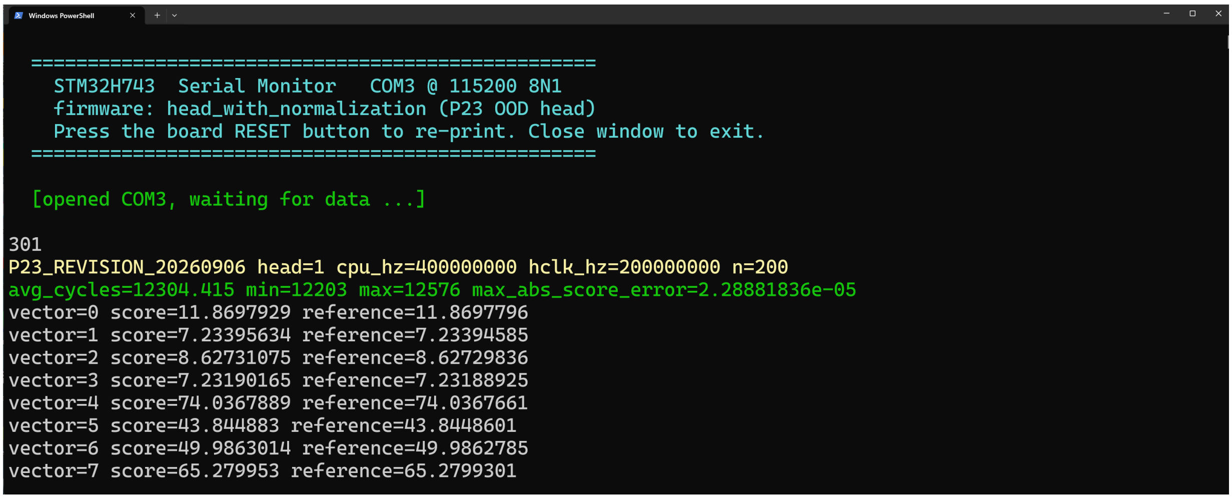

Figure S2: Live serial-monitor capture (COM3 @ 115200 baud, 8N1) of the running firmware reporting the 400 MHz DWT measurement. The highlighted line reports avg_cycles = 12304.415 over 200 calls (i.e. 30.76 us at 400 MHz) with max_abs_score_error = 2.29e-5 against the off-device reference over the eight embedded validation vectors.

S2 — On-chip measurement console log

The integer OOD head (firmware "head_with_normalization"; low-rank $r = 8$ int8 projection with fp32 scale assembly, 816 B of ROM constants) was flashed to the STM32H743 development board and run at 400 MHz. The DWT cycle counter measures the marginal head latency averaged over 200 calls; the eight embedded validation vectors check the integer kernel against the off-device reference. The firmware prints one block every 2 s; three consecutive blocks are reproduced verbatim below. Serial capture: COM3 @ 115200 baud, 8N1.

```
Supplementary Material S1 - On-chip measurement console log
Manuscript: biosensors-4534019 (PathoSafe)

The integer out-of-distribution head (firmware "head_with_normalization";
low-rank r = 8 int8 projection with fp32 scale assembly, 816 B of ROM
constants) was flashed to an STM32H743 development board (ATK-H743) and run
at 400 MHz. The DWT cycle counter measures the marginal head latency averaged
over 200 calls; the eight embedded validation vectors check the integer kernel
against the off-device reference. The firmware prints one block every 2 s;
three consecutive blocks are reproduced verbatim below to show repeatability.

Serial capture: COM3 @ 115200 baud, 8N1.

P23_REVISION_20260906 head=1 cpu_hz=400000000 hclk_hz=200000000 n=200
avg_cycles=12304.415 min=12203 max=12576 max_abs_score_error=2.28881836e-05
vector=0 score=11.8697929 reference=11.8697796
vector=1 score=7.23395634 reference=7.23394585
vector=2 score=8.62731075 reference=8.62729836
vector=3 score=7.23190165 reference=7.23188925
vector=4 score=74.0367889 reference=74.0367661
vector=5 score=43.844883 reference=43.8448601
vector=6 score=49.9863014 reference=49.9862785
vector=7 score=65.279953 reference=65.2799301

P23_REVISION_20260906 head=1 cpu_hz=400000000 hclk_hz=200000000 n=200
avg_cycles=12304.415 min=12203 max=12576 max_abs_score_error=2.28881836e-05
vector=0 score=11.8697929 reference=11.8697796
vector=1 score=7.23395634 reference=7.23394585
vector=2 score=8.62731075 reference=8.62729836
vector=3 score=7.23190165 reference=7.23188925
vector=4 score=74.0367889 reference=74.0367661
vector=5 score=43.844883 reference=43.8448601
vector=6 score=49.9863014 reference=49.9862785
vector=7 score=65.279953 reference=65.2799301

P23_REVISION_20260906 head=1 cpu_hz=400000000 hclk_hz=200000000 n=200
avg_cycles=12304.415 min=12203 max=12576 max_abs_score_error=2.28881836e-05
vector=0 score=11.8697929 reference=11.8697796
vector=1 score=7.23395634 reference=7.23394585
vector=2 score=8.62731075 reference=8.62729836
vector=3 score=7.23190165 reference=7.23188925
vector=4 score=74.0367889 reference=74.0367661
vector=5 score=43.844883 reference=43.8448601
vector=6 score=49.9863014 reference=49.9862785
```

```
vector=7 score=65.279953 reference=65.2799301

Mapping to the On-Chip Measurement table of the revised manuscript:
  avg_cycles = 12304.415 at 400 MHz  ->  30.761 us  (reported as 30.76 us)
  max_abs_score_error = 2.28881836e-05        (reported as 2.3e-5)
  head = 1   (normalization wrapper active)
  n = 200    (iteration count of the latency average)
```

Mapping to the On-Chip Measurement table (Table 11) of the revised manuscript:

- avg_cycles = 12304.415 at 400 MHz → 30.761 us (reported as 30.76 us)
- max_abs_score_error = 2.28881836e-05 (reported as 2.3e-5)
- head = 1 (normalization wrapper active)
- n = 200 (iteration count of the latency average)
